# Saponins from *Oxybasis rubra* (L.) S.Fuentes, Uotila & Borsh: Comparative Assessment of Cytotoxic Potential Against a Wide Panel of Cancer Cell Lines

**DOI:** 10.3390/molecules30153126

**Published:** 2025-07-25

**Authors:** Karolina Grabowska, Adam Mynarski, Agnieszka Galanty, Dagmara Wróbel-Biedrawa, Paweł Żmudzki, Irma Podolak

**Affiliations:** 1 Chair of Pharmacognosy, Jagiellonian University Medical College, 9 Medyczna Str., 30-688 Cracow, Poland; karolina1.grabowska@uj.edu.pl (K.G.); adammynar44@o2.pl (A.M.); agnieszka.galanty@uj.edu.pl (A.G.); dagmara.wrobel-biedrawa@uj.edu.pl (D.W.-B.); 2 Department of Medicinal Chemistry, Jagiellonian University Medical College, 9 Medyczna Str., 30-688 Cracow, Poland; pawel.zmudzki@uj.edu.pl; 3 Center for the Development of Therapies for Civilization and Age-Related Diseases, Jagiellonian University Medical College, Skawińska 8, 31-066 Krakow, Poland

**Keywords:** triterpene saponins, *Oxybasis rubra*, isolation, cytotoxicity, structure-activity observations, multivariate analysis, Amaranthaceae

## Abstract

Two triterpene saponins, hederagenin glucosides, including a novel monodesmoside: 3-*O*-β-D-glucopyranosyl(1→3)-β-D-glucopyranosyl] hederagenin (compound **1**), were isolated from the fruits of *Oxybasis rubra* (L.) S.Fuentes, Uotila & Borsh (Amaranthaceae). These compounds, together with hederagenin itself (compound **4**) and a commercially available 28-*O*-β-D-glucopyranosyl hederagenin ester (compound **3**), were evaluated for cytotoxicity and selectivity across a wide panel of human cancer cell lines (skin, prostate, gastrointestinal, thyroid, and lung). All four compounds exhibited dose- and time-dependent effects, with varying potency depending on the specific cancer type. The isolated bidesmosidic saponin (3-*O*-β-D-glucopyranosyl(1→3)-β-D-glucopyranosyl] hederagenin 28-*O*-β-D-glucopyranosyl ester—compound **2**) showed the strongest activity and selectivity, with an IC_50_ = 6.52 μg/mL after 48 h incubation against WM793 melanoma, and almost no effect on normal HaCaT skin cells (IC_50_ = 39.94 μg/mL). Multivariate analysis of the obtained data using principal component analysis (PCA) and hierarchical cluster analysis (HCA) supported the assumption that cytotoxicity is influenced by the type of compound, its concentration, and the intrinsic sensitivity of the cell line. Structure-activity observations between closely related hederagenin derivatives are also briefly presented.

## 1. Introduction

Screening studies on cytotoxic activity of compounds against cancer cell lines, initiated by the National Cancer Institute (NCI) in the 1960s, represent a first, very important step in the discovery of new oncological therapeutics. Each year, a vast number of plant extracts, and compounds isolated from them, are subjected to such evaluations. Species selection plays a pivotal role in this process, and one of the most frequently chosen strategies, with a high percentage of success, involves ethnopharmacological selection—choosing plants based on their previous traditional or folk medicinal use. Notably, a significant proportion of currently used anticancer drugs have entered clinical practice as a result of this approach to plant selection [1,2].

*Oxybasis rubra* (L.) S.Fuentes, Uotila & Borsh (syn. *Chenopodium rubrum* L., Red goosefoot), a member of the Amaranthaceae family, was reclassified into the genus *Oxybasis* only in 2012 [3]. The plant has been used in traditional Ayurvedic and folk medicine, with some reports indicating its application in the treatment of inflamed tumors [4]. It was also valued as a dietary component by the Gosiute Indians in Utah, and used as a natural dye [5,6]. *O. rubra* is found in both natural and synanthropic habitats, primarily within temperate biomes, and is widely distributed across North America and Eurasia [7,8,9]. As the plant readily adapts to different growth conditions [10], it can be easily harvested for medicinal use or as a source of bioactive compounds. Botanically, *O. rubra* is an annual herbaceous plant ranging in height from a few centimeters to approximately one and a half meters, with green or reddish stems, that are erect or branched. The leaves are triangular to ovate, with large blunt lobes and notched margins, very variable in size and shape. A distinguishing characteristic is their glossy surface. The flowers are reddish, and the seeds are brown [9].

Phytochemical data on *O. rubra* remains limited. Existing reports indicate the presence of essential oil, phenolics (flavonoids and phenolic acids), phytosterols, melatonin, and betalains—the latter also identified in in vitro plant cultures [11,12,13,14,15,16]. In our recent study, we additionally detected minor quantities of triterpene saponins, specifically chikusetsusaponin IVa and calenduloside E across all examined plant parts [17].

Despite the documented traditional use of the plant, little is known about the specific compounds responsible for its folk medicinal properties. Single reports refer to the antioxidant effects and cytotoxic activity of complex extracts against individual cancer cell lines, such as lung—A549, colon—HT15, HT29, and neuroblastoma—IMR32 [15,18]. In our previous preliminary study, methanolic extracts from the fruits demonstrated significant cytotoxic activity against the WM793 skin cancer cell line [16]. These findings, together with reports on the traditional use of *O. rubra*, encouraged us to investigate the bioactive constituents responsible for the observed cytotoxic effects.

According to chemotaxonomic data, the Amaranthaceae family is characterized by a distinct phytochemical profile, with the presence of triterpene saponins being a typical feature [19,20]. Furthermore, there are several reports on the cytotoxic potential of saponins isolated from botanically related species, for example *Chenopodium quinoa*, *Ch. album*, *Ch. bonus-henricus*, *Ch. foliosum*, *Ch. hybridum*, and *Ch. strictum*. These findings suggest that this class of plant metabolites may also contribute to the biological activity observed in *O. rubra* [21,22,23,24,25,26,27].

Saponins are natural glycosidic compounds that have long attracted interest due to their diverse therapeutic potential. These metabolites are characteristic of many plant species, and are often responsible for their medicinal properties, as exemplified by ginseng, horse chestnut or licorice [28]. A substantial body of literature documents the pharmacological activity of saponins—particularly those of the triterpene type—including anti-inflammatory, immunomodulatory, adaptogenic, hypoglycemic, chemosensitizing, antiviral, and antibacterial. Notably, a significant proportion of these studies focus on their cytotoxic activity, both in vitro and in vivo [29,30,31]. According to our recent review, approximately 40 novel triterpene saponins with potent cytotoxic effects (IC_50_ < 10 µM) are reported annually [32]. The antitumor activity reported for saponins is multifaceted, involving various mechanisms, that ultimately lead to cell death. While apoptosis stimulation and nonapoptotic cell death pathways are most commonly implicated, recent data have also highlighted alternative mechanisms, such as ferroptosis, anoikis, necroptosis or methuosis [32].

The majority of cytotoxically active saponins represent the triterpene type, and extensive experimental evidence confirms that their activity is influenced by both the structure of the aglycone moiety and the composition of the sugar chains [32,33] Given that even subtle changes in the structure of chemical molecules can translate into differences in the activity profile (as exemplified by the clinically used anticancer alkaloids such as vincristine and vinblastine, which differ by only a single substituent), it is particularly important to investigate structurally related compounds with a focus on structure-activity observations. Saponins reported from various species within the Amaranthaceae family represent diverse structural types, making them an ideal object of comparative studies [19,25].

Thus, the goal of the current study was to isolate saponins from the bioactive extract of *O. rubra* fruits, elucidate the structures of the major compounds, and evaluate their cytotoxic activity across a broad panel of human cancer cell lines representing the most prevalent neoplasms (skin, prostate, thyroid, lung, GI) varying in the degree of malignancy. To assess selectivity, the effects on normal human cells were also examined. The resulting data were subjected to an in-depth multivariate statistical analysis to enable comparative evaluation of cytotoxic effects in relation to both cell line sensitivity and specific structural features of the individual saponins.

## 2. Results and Discussion

### 2.1. Isolation and Structure Elucidation of Saponins

Crude dried methanolic extract from deflated fruits of *Oxybasis rubra* was dissolved in water and eluted with *n*-butanol to obtain saponin-rich fraction. Subsequent multistep chromatographic procedures with different experimentally optimized stationary (NP and RP) and mobile phases, involving mainly open column chromatography (CC), medium-pressure liquid chromatography (MPLC), and preparative thin-layer chromatography (pTLC), yielded two compounds **1** (24 mg) and **2** (84 mg). Both gave a positive Liebermann-Burchard test, were isolated in purity of over 95%, as confirmed by HPLC with ELSD detection, and were subjected to analyses leading to structure elucidation. 

**Figure 1:**
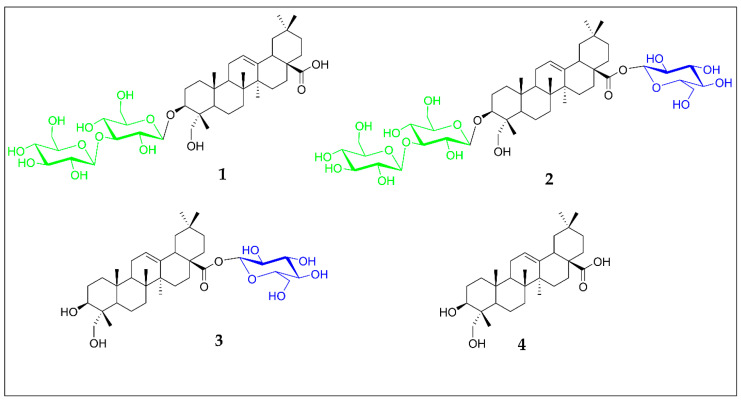
Structures of saponins **1** (3-*O*-β-D-glucopyranosyl(1→3)-β-D-glucopyranosyl] hederagenin) and **2** (3-*O*-β-D-glucopyranosyl(1→3)-β-D-glucopyranosyl] hederagenin 28-*O*-β-D-glucopyranosyl ester) isolated from *Oxybasis rubra*, together with 28-*O*-β-D-glucopyranosyl hederagenin ester (**3**) and hederagenin (**4**).

**Figure 2:**
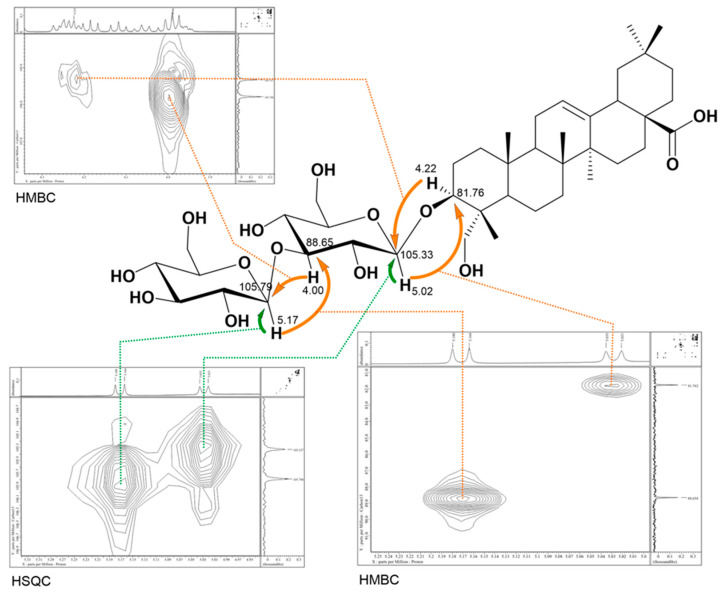
Key correlations (HMBC and HSQC) with regard to the sugar chain of compound **1.** The corresponding spectra are presented in the Supplementary Materials file (Figures S3 and S4).

Compound **1** (Figure 1) was obtained as a white amorphous powder. The molecular formula was established based on the HR-ESI-MS spectrum showing a peak at *m*/*z* 819.4507 [M + Na]^+^ calculated for C_42_H_68_O_14_Na (Figure S10). Acid hydrolysis of compound **1** on a TLC plate and comparison with sapogenin and sugar standards indicated the presence of hederagenin and glucose (Figures S23 and S24). The ^1^H NMR and ^13^C NMR spectra of **1** (Table 1, Figures S1 and S2) together with HSQC (Heteronuclear Single Quantum Coherence) spectra (Figure S3) allowed to establish signals typical of an oleane-12-ene type of sapogenin, with a *triplet* of the olefinic proton (δ 5.40, H-12), and olefinic carbon signals of the double bond at δ 122.29 (C-12) and 144.78 (C-13). ^13^C signals of six tertiary methyl groups at δ 13.53, 15.88, 17.32, 23.61, 26.01, and 33.09 were assigned, respectively, to Me-24 (δ 0.89, *s*, 3H), Me-25 (δ 0.84, *s*, 3H), Me-26 (δ 0.95, *s*, 3H), Me-29 (δ 0.93, *s*, 3H), Me-27 (δ 1.19, *s*, 3H), and Me-30 (δ 0.86, *s*, 3H). The signal at δ 180.37 pointed to a carbonyl carbon at C-28, as indicated by correlations observed in HMBC (Heteronuclear Multiple Bond Correlation) spectrum (Figure S4), between H-18 (δ 3.24, *dd J* = 13.7 and 4.0 Hz) and C-28, while the substitution of C-23 with a hydroxyl was clearly seen from the carbon downfield shift (δ 64.04) and signals of two protons in HSQC at δ 3.66 and 4.26. The above data were in good agreement with hederagenin, glycosylated at C-3 (as confirmed by a downfield C-3 shift at δ 81.76, and a characteristic oxymethine proton shift at δ 4.22 (*dd*, *J* = 11.8 and 4.4 Hz,) [Liu 2022 [34]]. The positive-ion electrospray ionization mass spectrometry (ESI-MS) of compound **1** displayed ion peaks at *m*/*z* 635.63 and *m*/*z* 473.29 corresponding to the loss of one hexose and then the second hexose, respectively (Figure S11). The monosaccharide composition and interglycosidic linkage within the sugar chain were further elucidated based on detailed analysis of spectra from 2D NMR experiments: ^1^H-^1^H 2D COSY (Correlation Spectroscopy) (Figure S6), TOCSY (Total Correlation Spectroscopy) (Figure S7), ROESY (Rotating-frame Overhauser Enhancement Spectroscopy) (Figure S8), ^1^H-^13^C HMBC, H2BC (Heteronuclear two-bond Correlation) (Figure S5), and HSQC. Accordingly, two *doublets* of anomeric protons at δ 5.02 *(J* = 7.0 Hz) and 5.17 (*J* = 7.5 Hz) which corresponded to the carbon signals at δ 105.33 and 105.79 have been assigned to two β-glucopyranoses, with a terminal glucose (Glc’) linked at C-3 of glucose (Glc) directly attached to the C-3 of hederagenin. This was established from the HMBC correlation between its anomeric proton at 5.17 and C-3 of Glc (δ 88.65) while the crosspeak between the anomeric proton of Glc at δ 5.02 and the aglycone carbon signal at δ 81.76 (C-3) confirmed the sugar chain attachment (Figure 2). Consequently, the structure of compound **1** was elucidated as 3-*O*-β-D-glucopyranosyl(1→3)-β-D-glucopyranosyl] hederagenin, which is a new saponin, previously not reported in literature.

**Figure 3:**
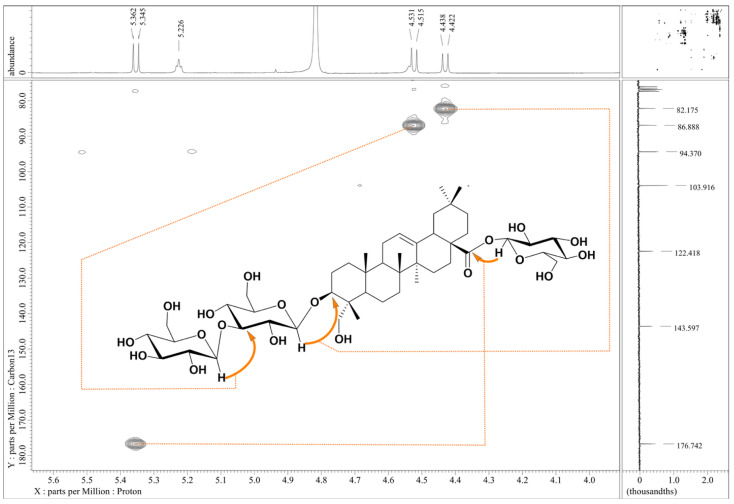
Key HMBC correlations of compound **2**.

The HR-ESI-MS data of compound **2** (a peak at *m*/*z* 981.5035 [M + Na]^+^ calculated for C_48_H_78_O_19_Na) indicated the presence of an additional hexose, compared to compound **1** (Figure S15). Acid hydrolysis of **2** on a TLC plate gave hederagenin and glucose (Figures S23 and S24). Positive-ion electrospray ionization mass spectrometry (ESI-MS) of compound **2** displayed ion peaks (*m*/*z* 797.72, *m*/*z* 635.56, *m*/*z* 473.55, indicating the presence of three hexoses in the molecule (Figure S16). Then, careful examination of 1D and 2D NMR data of **2** (Figures S12–S19) showed its close resemblance to **1** except for the glycone composed of three β-glucopyranoses, with one monosaccharide attached at C-28. This was seen from the upfield shift in its anomeric carbon (δ 94.37) (see Table S1) and HMBC correlation between the anomeric H-1 (δ 5.35) and the carbon signal at δ 176.74 (C-28) (see Figure 3). Thus, the structure of compound **2** was established as 3-*O*-β-D-glucopyranosyl(1→3)-β-D-glucopyranosyl] hederagenin 28-*O*-β-D-glucopyranosyl ester.

Literature data referring to previous reports on this bidesmosidic saponin structure are ambiguous. It has been listed as one of the constituents of *Ixeris sonchifolia* (Asteraceae) in two recent and one earlier review papers focused on this interesting TCM plant species, which has a long history across several Asian countries, particularly China, and a broad spectrum of contemporary reported pharmacological activities [35,36,37]. However, no comparative spectroscopic evidence for this saponin has been published in English-language journals. The original source describing its isolation and structure elucidation appears to be a PhD dissertation by Feng X. Z., available only in Chinese, and thus considered grey literature [38]. Li cited this dissertation and referred to the compound as Ixeris saponin C [37]. Zhang [35], in his review, also provides the same information as Li [37], although citing the study by Feng published in Planta Medica in 2003. However, in that publication on saponins from *I. sonchifolia*, Feng describes structure elucidation of Ixeris saponin C, presenting under this name a compound with a different sugar chain at C-3, namely 3-*O*-[β-D-glucopyranosyl(1→3)-β-D-glucopyranosyl(1→3)-α-L-arabinopyranosyl]-16α,23-dihydroxyolean-12-ene 28-*O*-β-D-glucopyranosyl ester [39]. The reference later cited by Wen [36] in his review pertains to another work by Feng, which does not directly address the structure elucidation of this saponin. To sum up, although the structure of compound **2** has been mentioned in previous reviews, to our knowledge, this is the first complete spectroscopic characterization published in a peer-reviewed journal.

Saponins reported to date in the Amaranthaceae family include glycosides of various oleanane-type sapogenins, such as oleanolic acid and its hydroxy-derivatives, gipsogenin, saikogenins, sophradiol, phytolaccagenic, akebenoic, spergulagenic, medicagenic, and serjanic acids, as well as hederagenin. Notably, both compounds **1** and **2** isolated in this study from *O. rubra*, represent structures that have not previously been reported in either the *Oxybasis* genus or the broader Amaranthaceae family [19,20,24,25,26,27]. Furthermore, although hederagenin-type saponins are widely distributed across various plant species within different families, the presence of a bi-glucose sugar chain with a (1→3) linkage is rare [40].

### 2.2. Cytotoxicity Screening

Building on our previous report on the cytotoxic activity of crude extract from the fruits of *O. rubra* [16] as well as many studies highlighting the activity of various hederagenin-type saponins [41,42,43], we decided to check the cytotoxic potential of the two isolated compounds bearing a distinctive 3-*O*-β-D-glucopyranosyl-(1→3)-β-D-glucopyranosyl sugar chain. To gain deeper insight into how subtle structural differences influence the overall activity, we included in the cytotoxicity assay not only compounds **1** and **2** but also a commercially available 28-*O*-β-D-glucopyranosyl hederagenin ester (compound **3**) and hederagenin itself (compound **4**) (Figure 1).

All four structurally related compounds were screened for cytotoxic activity against a broad panel of human cancer cell lines of varying origin, malignancy, and metastatic potential (skin, prostate, gastrointestinal, thyroid, lung). The cytotoxic effect was evaluated using the LDH colorimetric test, with doxorubicin serving as the reference drug. Selectivity was assessed with respect to normal cell lines (PNT2 and HaCaT). Data expressed as IC_50_ is summarized in Table 2 and Table S2.

The results demonstrated that the tested compounds exhibited varying activity and selectivity, depending on their structural features and the sensitivity of the cancer cell lines. All compounds acted in a dose- and time-dependent manner, with more pronounced effects observed after 48 h of incubation compared to 24 h.

Among the tested compounds, saponin **2** showed the strongest cytotoxic activity and selectivity, with an IC_50_ = 6.52 μg/mL after 48 h incubation against WM793 melanoma, while exhibiting minimal toxicity towards normal HaCaT skin cells IC_50_ = 39.94 μg/mL. Its efficacy was also significant against the lung cancer cell line A549 (IC_50_ = 8.24 μg/mL after 48 h). Notably, compound **2** was non-toxic to normal prostate epithelial cells, while it showed moderate toxicity against DU-145. Compound **1** exhibited activity against all cell lines tested whereas compound **4** (hederagenin) was generally inactive. Hederagenin, a triterpenoid widely studied for its potential anticancer properties, has been reported to exhibit significant cytotoxic effects, particularly in its semi-synthetic derivatives, as summarized in several recent reviews [44,45,46]. The weak activity of hederagenin observed in our study may be attributed to cell line specificity and different susceptibility, as hederagenin has not previously been tested against most of the cell lines included in our panel. Although the A549 lung cancer cell line is among the most frequently used in cytotoxicity screening, reported IC_50_ values for hederagenin vary considerably across studies—ranging from 11 to >100 μM—likely due to differences in experimental methodology [44,45,46].

### 2.3. Multivariate Analysis of Cytotoxicity Screening Results

**Figure 4:**
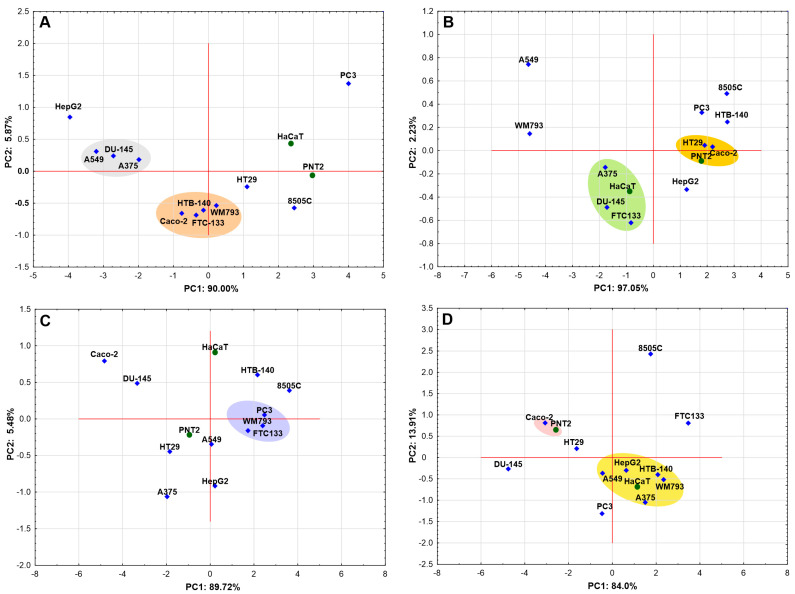
PCA score scatterplots (**A**–**D**) showing the projection of cases onto the first two principal components (PC1 and PC2). Each panel (**A**–**D**) corresponds to a specific compound: (**A**)—compound **1** (3-*O*-β-D-glucopyranosyl(1→3)-β-D-glucopyranosyl] hederagenin); (**B**)—compound **2** (3-*O*-β-D-glucopyranosyl(1→3)-β-D-glucopyranosyl] hederagenin 28-*O*-β-D-glucopyranosyl ester); (**C**)—compound **3** (28-*O*-β-D-glucopyranosyl hederagenin ester); (**D**)—compound **4** (hederagenin). Blue diamonds indicate cancer cell lines, while green circles represent normal (non-cancerous) cell lines. Colored ellipses indicate grouping tendencies. Abbreviations: HTB-140: malignant melanoma, A375: malignant melanoma, WM793: primary melanoma, HaCaT: normal keratinocytes, DU-145: metastatic prostate carcinoma, PC3: metastatic prostate carcinoma, PNT2: prostate epithelial cells, FTC133: follicular thyroid carcinoma, 8505C: undifferentiated thyroid carcinoma, Caco-2: colon adenocarcinoma, HT29: colon adenocarcinoma, HepG2: hepatocellular carcinoma, A549: lung carcinoma.

Multivariate analysis, including Principal Component Analysis (PCA) and Hierarchical Cluster Analysis (HCA), was employed to explore the similarities in the effects of the four compounds, tested at various concentrations, across different cell lines. Each of the PCA models constructed met the necessary validity criteria, including an adequate KMO (Kaiser-Meyer-Olkin Measure of Sampling Adequacy) value (≥0.729) and statistical significance in the Bartlett test (*p* < 0.05). The models explained a sufficiently large proportion of the variance > 92% in the dataset. To illustrate whether the cell lines show a similar pattern of response (sensitivity) to a given compound, a PCA model was constructed for each compound. The resulting factor score plots, shown in Figure 4, illustrate the distribution of cases (cell lines) in factor space (PC1, PC2), enabling visualization of interrelationships.

PCA analysis revealed two distinct groups of cell lines for compound **1** (Figure 4A): one consisting of cell lines A375, A549, and DU-145, and the other comprising WM793, Caco-2, HTB-140, and FTC133. Each of these groups shares a distinct sensitivity pattern to compound **1**. Cell lines located far apart indicate different sensitivity to the compound analyzed. In the case of compound **2**, one distinct group of three cell lines (HT29, Caco-2, PNT2) forms a clear cluster, while a looser, less compact grouping can also be observed (Figure 4). This suggests a similar but not uniform pattern of response among these cell lines. PCA analysis also shows that the PC3, FTC133, and WM793 cell lines exhibited a consistent response pattern to compound **3**. Also, for compound **4**, the cell lines form the groups as can be seen in Figure 4.

It is noteworthy that in the analyses performed, a clear pattern of cell line grouping emerges for each substance analyzed, thus indicating that the cell lines show different sensitivity to the compounds tested. Unfortunately, for none of the substances was a pattern leading to a clear separate clustering of cancer cell lines and normal cell lines observed, suggesting a lack of selectivity of the analyzed compounds. Although PCA analysis for compound **2** did not reveal distinct clustering of non-cancerous cell lines, cytotoxicity assays indicated that the compound was inactive against normal prostate epithelial cells (IC_50_ > 100 µg/mL) and showed only low activity against normal HaCaT skin cells (IC_50_ = 39.94 µg/mL). For this reason, compound **2** was classified as selective. To investigate whether there are general but hidden patterns of behavior regarding the sensitivity of the tested cell lines to all compounds analyzed, an attempt was made to create a hierarchical PCA model (hPCA) using the first two principal components (PC1, PC2) of each individual simple PCA model (created for each compound). However, the low KMO coefficient (0.368) and high *p*-value in the Bartlett test (*p* = 0.53) for this data set suggest that the data do not have sufficient correlation structure for hPCA to be effective, supporting the assumption that the sensitivity of cell lines to the compounds tested varies and no common overall sensitivity pattern can be found.

Therefore, to further explore potential similarities in the effects of the tested compounds at different concentrations on cancer cell lines, a PCA model was constructed using the cancer cell lines as variables and the compounds at various concentrations as cases. In this model, the first principal component (PC1) accounted for 80.93% of the variability while the second component (PC2) explained an additional 11.79%, together capturing 92.72% of the total variance. The key features of the model are shown in Table 3.

**Figure 5:**
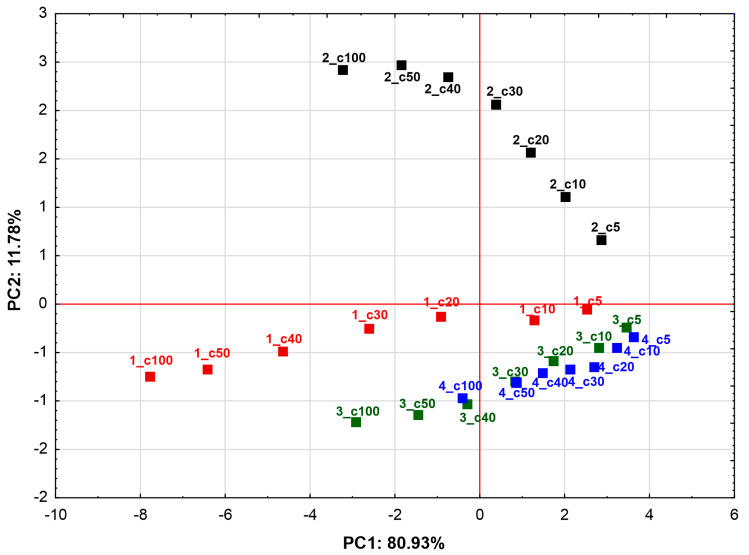
The score scatterplot of PCA model based on first two principal components (PC1 and PC2). Colored squares represent different samples (compounds tested at varying concentrations). Colors denote compounds as follows: red—compound **1** (3-*O*-β-D-glucopyranosyl(1→3)-β-D-glucopyranosyl] hederagenin); black—compound **2** (3-*O*-β-D-glucopyranosyl(1→3)-β-D-glucopyranosyl] hederagenin 28-*O*-β-D-glucopyranosyl ester); green—compound **3** (28-*O*-β-D-glucopyranosyl hederagenin ester); blue—compound **4** (hederagenin). Each sample is labelled with a code in the format Z_cX, where Z indicates the compound number (1–4) and cX specifies the concentration in μg/mL. For example, 1_c50 corresponds to compound 1 at 50 μg/mL.

The scatter plot of the case projections on the principal component planes (Figure 5) revealed a clear grouping of the data depending on the compounds tested and illustrated the effect of increasing compound concentration on cytotoxicity against cell lines. The graph shows a spatial separation of scores for compound **1** and compound **2** and a common grouping trend of cases for compounds **3** and **4**. This clearly indicates that compounds **1** and **2** differ from each other and from compounds **3** and **4** in their activity profile towards cell lines. These differences are particularly apparent in the higher concentration range and may be due, among other things, to the different chemical structure of the analyzed substances. Compound **1** is a monodesmoside, while compound **2** differs from it only by the presence of an additional glucose moiety (attached via the C-17 (28-COOH) position). It is worth noting that both compounds, unlike compounds **3** and **4**, have a sugar chain linked at the C-3 position of hederagenin. In contrast, compounds **3** and **4** have a free hydroxyl group at the C-3 position. The results obtained in the PCA model suggest that these differences in structural features may influence the pattern of activity of the compounds against cell lines.

**Figure 6:**
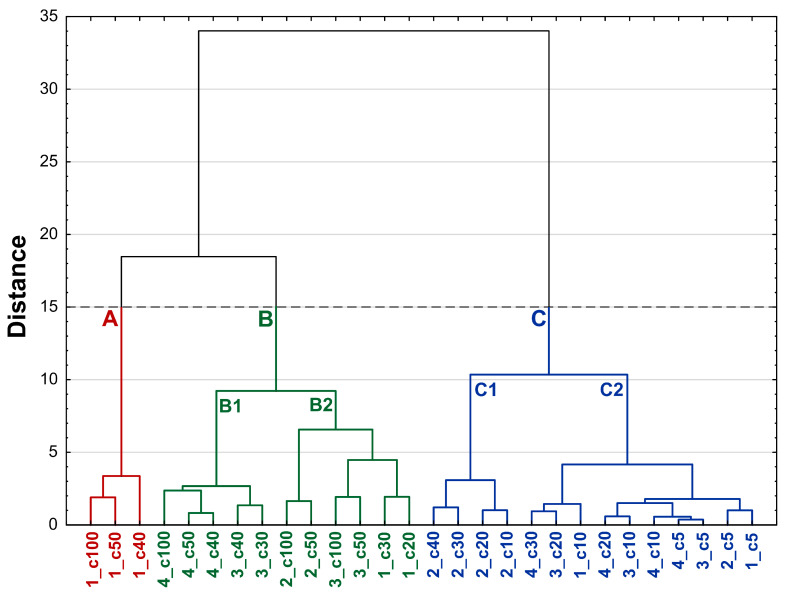
Dendrogram of similarity among investigated compounds **1–4** at different concentrations (dashed horizontal line indicates that the grouping has been stopped according to Mojena’s rule). Clusters were marked with subsequent letters: A, B, C, and distinguished by colors (A—red; B—green; C—blue). Each sample is labelled with a code in the format Z_cX, where Z indicates the compound number (**1–4**) and cX specifies the concentration in μg/mL. For example, 1_c50 corresponds to compound **1** at 50 μg/mL. Abbreviations: 1—compound **1** (3-*O*-β-D-glucopyranosyl(1→3)-β-D-glucopyranosyl] hederagenin); 2—compound **2** (3-*O*-β-D-glucopyranosyl(1→3)-β-D-glucopyranosyl] hederagenin 28-*O*-β-D-glucopyranosyl ester); 3—compound **3** (28-*O*-β-D-glucopyranosyl hederagenin ester); 4—compound **4** (hederagenin).

The PCA analysis showed a trend in the effects of the analyzed substances in terms of their overall effect on cell lines. Therefore, to investigate and classify which compounds at which concentration show similar effects on cell lines, a hierarchical cluster analysis (HCA) with Ward’s grouping method and Euclidean distance was performed. According to Mojena’s rule, the substances were divided into three main clusters: A, B, C (Figure 6). The analysis showed that the observed cytotoxic effect depends not only on the compound used, but also on its specific concentration. Cluster A included solely different concentrations of compound **1** (40–100 μg/mL), showing significantly higher cytotoxicity against all analyzed cell lines compared to the other compounds tested, irrespective of their concentration. The next separated multi-element clusters B and C contain substances at concentrations with lower cytotoxic activity against cell lines than those in cluster A. Within cluster B, two subclusters can be distinguished: B1 and B2, the latter of which groups compounds at concentrations with higher cytotoxicity against cell lines: A375, A549, HepG2, FTC133, and WM793 than cluster B1. The next cluster C contains compounds at concentrations with the lowest activity against most of the cell lines tested. It is noteworthy, however, that cluster C includes subcluster C1, which contains only compound **2** (at concentrations of 10–40 μg/mL) with significantly higher activity against lines A549 and WM793 compared to the other concentrations of substances in this cluster. Cluster analysis showed that compounds **3** and **4** occur together in the same subclusters (B1). In contrast, compound **4** and compound **2** at concentrations of 10–100 μg/mL do not occur together in the same subclusters. These observations are consistent with the PCA model. Furthermore, the results from the PCA and HCA models supported the assumptions that the observed cytotoxicity depends on the type of compound, its concentration, and the sensitivity of the cell line.

### 2.4. Structure-Activity Observations

Comparative analysis of the cytotoxicity assay results for the four structurally closely related compounds allows for some preliminary observations regarding the relationship between the structure and activity. While a general pattern, or a trend in the behavior of compounds towards cell lines, was evident in the PCA models, HCA analysis confirmed that structurally distinct compounds may exhibit similar effects depending on concentration. Therefore, to accurately detect relationships between compound structure and cytotoxicity, it is essential to analyze each cell line individually and compare the effects at specific concentrations. A considerable number of studies have investigated the role of a free carboxyl group at the C-17 position of the aglycone (28COOH) as a structural feature contributing to the cytotoxic activity of the pentacyclic triterpenoids, particularly oleanane derivatives [32]. The results of the present study suggest that the absence of a free COOH group in the structure of the compound, may also influence the activity, particularly in the case of saponins. However, the presence of a sugar moiety appears to be the primary determinant of cytotoxic activity in terpenoids, including hederagenin (HE) derivatives. A comparison of the activity of HE (compound **4**) and HE-type saponins (**1**, **2**, **3**) indicates that the lack of a sugar moiety in the molecule significantly reduces or abolishes cytotoxic activity against human cell lines (Table 2). Moreover, the position and structure of the sugar chain are critical for potency. The results obtained in our study indicate that hederagenin monodesmoside (compound **1**), in which the sugar moiety was attached to the C-3 of the aglycone, exhibited the highest cytotoxic activity across the broadest range of tested cell lines.

When considering the structure of the two HE-type monodesmosidic saponins (compounds **1** and **3**) evaluated in the current study, it should be noted that the blockage of the C-17 carboxyl group (28COOH) with a sugar chain (as in 3) practically eliminated the activity against HTB-140, WM793, PC3, FTC133, 8505C cells, and decreased the effect against A375, DU-145, PNT2, HT29, HepG2. Furthermore, the presence of a second sugar chain located at C-3 of hederagenin while blocking 28COOH via glucose in compound **2** increased its effect against WM793 and A549 relative to that induced by compound **3**. Interestingly, the cytotoxic effect of the analysed bidesmoside (compound **2**) against the A549 line was comparable to that of the corresponding monodesmoside (compound **1**), and even higher in the case of the WM793 line. These observations suggest that for the action of HE-type saponins against these cell lines, it is not the presence of the free carboxyl group 28COOH that is crucial, but the specific structure of the sugar chain in position C-3. Moreover, in the case of WM793 cells, the presence of an additional, ester-attached glucose (via 28COOH) increased the activity of bidesmoside (compound **2**) compared to the corresponding monodesmoside (compound **1**). This is consistent with other studies recently published on oleanane derivatives [47].

## 3. Materials and Methods

### 3.1. Chemicals and Reagents

Chloroform, ethyl acetate, methanol, *n*-butanol, 2-propanol, acetone, aniline phthalate, potassium chloride, and sulfuric acid, were from CHEMPUR (Gliwice, Poland). All reagents were of analytical grade. Hederagenin, oleanolic acid (purity ≥ 97.0%, HPLC), D-glucose, D-galactose, L-arabinose, D-xylose, glucuronic acid, HPLC-grade acetonitrile and formic acid, were from Sigma-Aldrich (St. Louis, MO, USA). Hederagenin 28-*O*-B-D-glucopyranosyl ester (purity ≥ 98.0%, HPLC) was from ChemNorm Biotech Co. Ltd. (Wuhan, China). HPLC-grade methanol and acetonitrile were from Merck (Darmstadt, Germany). Methanol-*d*_4_ and pyridine-*d*_5_ for NMR analyses were from Thermo Scientific (Acros, Switzerland). Water was prepared using a Milli-Q system (Millipore Corp., Bedford, MA, USA).

### 3.2. General Experimental Procedures

Open column chromatography (CC) was carried out on NP: silica gel (230–400 mesh; Sigma-Aldrich, Darmstadt, Germany) using a glass column (350 × 25 mm). For medium-pressure liquid chromatography (MPLC), a Sepacore apparatus equipped with a C-615 Pump Manager (BÜCHI Labortechnik AG, Flawil, Switzerland) was used. MPLC was carried out on NP—silica gel 230–400 mesh (Sigma-Aldrich, Germany) and RP: reverse phase silica gel (LiChroprep, RP-18 (40–63 μm), Merck, Darmstadt, Germany). Preparative thin-layer chromatography (pTLC) was conducted on Silica Gel G (500 μ) ANALTECH (Miles Scientific, Newark, DE, USA). Detection: spraying with water. For analytical thin-layer chromatography (TLC) silica gel 60 plates (NP) (Merck, Germany) and RP-18 F_254_S silica gel 60 plates (Supelco, Darmstadt, Germany) were used. Detection: spraying with 25% solution of H_2_SO_4_ in methanol and heating at 120 °C for 4 min. on a TLC plate heater (CAMAG, Muttenz, Switzerland) for visualization.

The HPLC-PDA-ELSD system (Shimadzu, Kyoto, Japan) consisted of SLC-40 system controller, LC-40D XR solvent delivery module, DGU-405 degassing unit, SIL-40C XR auto sampler, CTO-40C column oven, SPD-M40 photo diode detector, and ELSD-LT III low temperature-evaporative light scattering detector. For chromatographic separations the ReproSil-Pur Basic-C18; 5 μm 250 × 4.6 mm column (Dr. Maisch HPLC GmbH; Ammerbuch-Entringen, Germany) equipped with ReproSil-Pur Basic-C18; 5 μm 5 × 4.6 mm pre-column (Dr. Maisch HPLC GmbH; Germany) was used. Separation conditions: eluent A: water/formic acid (0.1%, *v*/*v*); eluent B: acetonitrile/formic acid (0.1%, *v*/*v*); gradient elution: eluent A 60%, eluent B 40% (0–9 min); eluent B 40% to 85% (9–12 min) eluent A 15%, eluent B 85% (12–14 min); eluent B 85% to 40% (14–15 min);.eluent A 60%, eluent B 40% (15–17 min); temperature: 30 °C; flow rate 1 mL/min; injection volume 10 μL. The ELSD set of parameters was as follows: the temperature of the ELSD drift tube: 35 °C; nebulizing gas: compressed air; and the signal sensitivity gain: wide. LabSolution Shimadzu Corporation software (version 5.124 SP1, Shimadzu, Kyoto, Japan) was used to record chromatograms.

LC-MS/MS analysis was carried out on UPLC/MS Waters ACQUITY TQD (Waters Corporation, Milford, MA, USA) apparatus operated in the negative and positive electrospray ionization mode. For chromatographic separations, the Acquity UPLC BEH (bridged ethyl hybrid) C18 column, 2.1 × 100 mm, and 1.7 µm particle size, equipped with Acquity UPLC BEH C18 VanGuard pre-column, 2.1 × 5 mm, and 1.7 µm particle size, was used. Separation conditions [gradient elution (95% to 0% of eluent A; eluent A: water/formic acid (0.1%, *v*/*v*); eluent B: acetonitrile/formic acid (0.1%, *v*/*v*)] and MS detection settings were described previously [26]. Chromatograms were recorded using Waters eλ PDA detector. Spectra were analyzed in 200–700 nm range with 1.2 nm resolution and sampling rate of 20 points/s.

HR-ESI-MS spectra for compounds **1** and **2** were recorded in positive-ion mode using Synapt G2-S HDMS (Waters Inc. USA) mass spectrometer equipped with an electrospray ion (ESI) source and q-TOF type mass analyzer. Samples were dissolved in methanol and injected directly into the electrospray ion source. Methanol was used as a solvent with a flow rate of 100 μL/min. The run time was 1 min. The instrument worked with external calibration on sodium formate in the mass range of *m/z* = 50–2000. The lock spray spectrum of the leucine-enkephalin was generated by the lock spray source, and correction was done for every spectrum. The exact mass measurements for all peaks were performed within 3 mDa mass error. The nitrogen was used as desolvation and cone gas, and their flow values were set to 580 L/h and 140 L/h, respectively. The desolvation gas flow temperature was set at 150 °C. The nebulizer gas pressure was set to 4.7 bar. The capillary voltage was set to 3.00 kV, and the sampling cone voltage and source offset were set to 20 V. The source temperature was 120 °C. The instrument was controlled, and recorded data were processed using the MassLynx V4.1 software package (Waters).

NMR experiments (1D (^1^H NMR—500 MHz, ^13^C NMR—125 MHz) and 2D (HSQC, H2BC, HMBC, COSY, TOCSY, ROESY) were performed on JNM-ECZR500 RS1 500 MHz (JEOL, Tokyo, Japan). Spectra for compound **1** were recorded in pyridinie-*d*_5_, for compound **2** in methanol-*d*_4_. Chemical shifts (δ) are given in ppm. Coupling constants are reported in Hz. Spectra were analyzed using JEOL Delta NMR processing and control software v5.3.3.

Melting points were determined using melting point apparatus Reichert (Austria). Cytotoxicity assay was performed using a Microplate Reader (BioTek Instruments Inc., Winooski, VT, USA) equipped with Gen 5 software.

### 3.3. Plant Material

The plant material—ripe fruits of *Oxybasis rubra* (L.) S.Fuentes, Uotila & Borsh, was collected at the fruiting stage of the plant (in September) from natural location in the village of Witów (50.14732853656126, 20.575107496284392) near Cracow, Poland. The species identity was confirmed by two independent botanists: Dr. Wacław Bartoszek PhD., Institute of Botany, PAN, and Sławomir Kasprzyk MSc. from the Department of Pharmacognosy, Jagiellonian University, Cracow, Poland. A voucher specimen (CR 2/09.2017) is kept at the Department of Pharmacognosy, Faculty of Pharmacy, Jagiellonian University Collegium Medicum, Cracow, Poland. The collected plant material was dried under controlled conditions (at 23 °C in air-conditioned room, in the dark) to a constant weight, ground to a fine powder with a mechanical laboratory mill (BOSCHMKM6003, BSHGmbH, Munich, Germany), and kept in airtight containers.

### 3.4. Extraction and Isolation

The dried fruits of *Oxybasis rubra* (200 g) were subjected to classical extraction (heat reflux extraction) with chloroform (3 × 3 h × 500 mL) followed by methanol (3 × 3 h × 600 mL). The methanol extracts were combined and evaporated to dryness under reduced pressure (rotary evaporator, 75 °C). The dry methanol extract (13.5 g) was suspended in water (110 mL) and extracted with n-butanol (2 × 2 h × 110 mL). The organic layer was pooled and evaporated using rotary evaporator. The dry residue (10.8 g) was separated by open column chromatography on silica gel (CHCl_3_/MeOH/H_2_O 23:12:2 *v*/*v*/*v*) to give 37 fractions (Fr_1_–Fr_37_) containing saponins which were combined based on a similar TLC profile (silica gel, CHCl_3_/MeOH/H_2_O 23:12:2 *v*/*v*/*v*, visualization: 25% methanolic H_2_SO_4_ + heating). Fractions rich in compound **1** were rechromatographed: Fr_5_ (85 mg) using CH_2_Cl_2_/MeOH/H_2_O (17:6:1 *v*/*v*/*v*); Fr_9_ (461 mg), Fr_10_ (274 mg), Fr_11_ (108 mg) using CHCl_3_/MeOH/H_2_O 25:10:0.5 *v*/*v*/*v*. This finally led to a pooled fraction containing compound **1** (84 mg) which was further purified (in four aliquots of 4 × 21 mg) using MPLC on RP-silica gel (Lichroprep RP-18 (40–63 μm); 150 × 12 mm column; flow rate 2.6 mL/min; and isocratic mode: methanol-water (7:2 *v*/*v*)). The resulting pooled fraction (36 mg) was finally purified by preparative TLC (silica gel, EtOAc/MeOH/H_2_O 24:3:2 *v*/*v*/*v*) to yield 24 mg of compound **1**. Compound **2** was isolated following successive chromatographic separation of Fr_15_ (150 mg), Fr_16_ (82 mg), Fr_17_ (325 mg) on silica gel (CHCl_3_/MeOH/H_2_O 23:12:2 *v*/*v*/*v*) to obtain pooled fractions: Fr.A‘ (32 mg) and Fr.B’ (192 mg). These were subjected to CC (silica gel EtOAc/MeOH/H_2_O 120:15:10 *v*/*v*/*v*) to eventually yield 84 mg of compound **2**. Purity was controlled using HPLC-ELSD technique (Figures S9 and S14) at conditions described in Section 3.2.

### 3.5. Acid Hydrolysis

Both isolated compounds **1** and **2** were subjected to acid hydrolysis on TLC plates with HCl *in statu nascendi* for 30 min at 60 °C. According to a procedure described previously [48], the plates were developed twice in CHCl_3_/MeOH/H_2_O (23:12:2 *v*/*v*) and visualized by spraying with aniline phthalate and heating (100 °C, 20 min) and analyzed with reference to sugar standards (D-glucose R_f_ = 0.32; D-galactose R_f_ = 0.28; L-arabinose R_f_ = 0.37; L-rhamnose R_f_ = 0.51; D-xylose R_f_ = 0.42; glucuronic acid lactone R_f_ = 0.60) (Figure S23). For analysis of sapogenins, the chromatogram was run once in CHCl_3_/EtOA*c* (4:1 *v*/*v*), visualized by spraying with 25% methanolic H_2_SO_4_ and heating (120 °C, 4 min), and analyzed with reference to sapogenin standards (hederagenin R_f_ = 0.13; oleanolic acid R_f_ = 0.55) (Figure S24).

### 3.6. Compounds

Compound **1**: 3-*O*-β-D-glucopyranosyl(1→3)-β-D-glucopyranosyl] hederagenin

White powder; (mp. 250–253 °C). ^1^H and ^13^C NMR: see Table 1. MS: HRESIMS *m*/*z* 819.4507 [M + Na]^+^ (calcd. for C_42_H_68_O_14_Na) (Figure S10);ESI (positive-ion mode) *m*/*z* 797.56 [M + H]^+^, *m*/*z* 819.6 [M + Na]^+^, fragmentation in MS/MS: *m*/*z* 635.63 [M+H-162]^+^, *m*/*z* 473.29 [M+H-162-162]^+^, *m*/*z* 455.2 [M+H-162-162-18]^+^, *m*/*z* 437.40 [M+H-162-162-18-18]; ESI MS (negative ion mode) *m*/*z* 795.45 [M-H]^−^, *m*/*z* 841.29 [M+45]^–^ (Figure S11). Acidic hydrolysis: sugar spot R_f_ = 0.32 (Figure S23), hederagenin spot R_f_ = 0.13 (Figure S24); Spectra are provided in the Supplementary Material (Figures S1–S8).

Compound **2**: 3-*O*-β-D-glucopyranosyl(1→3)-β-D-glucopyranosyl] hederagenin 28-*O*-β-D-glucopyranosyl ester

White powder; (mp. 220–223 °C). ^1^H and ^13^C NMR: see Table S1. MS: HRESIMS *m*/*z* 819.4507 [M + Na]^+^ (calcd. for C_42_H_68_O_14_Na) (Figure S21); ESI (positive-ion mode) *m*/*z* 959.9 [M + H]^+^, fragmentation in MS/MS: *m*/*z* 797.72 [M+H-162]^+^, *m*/*z* 635.56 [M+H-162-162]^+^, *m*/*z* 473.55 [M+H-162-162-162]^+^, *m*/*z* 455.54 [M+H-162-162-18]^+^ (Figure S22). Acidic hydrolysis: sugar spot. R_f_ = 0.32 (Figure S23), hederagenin spot R_f_ = 0.13 (Figure S24); Spectra are provided in the Supplementary Material (Figures S12–S19).

### 3.7. Cell Cultures and Cytotoxicity Assay

The cytotoxic activity was tested on human cancer and normal cell lines, grouped in several panels: skin (malignant melanoma HTB-140, malignant melanoma A375, melanoma WM793, and normal keratinocytes HaCaT), prostate (prostate carcinoma DU-145, prostate carcinoma PC3, and prostate epithelial cells PNT2), thyroid (thyroid carcinoma FTC133, thyroid carcinoma, undifferentiated 8505C), gastrointestinal panel (hepatocellular carcinoma HepG2, colon adenocarcinoma Caco-2, and colon adenocarcinoma HT29), and single line of lung carcinoma A549. Cells were grown at standard conditions (37 °C, 5% CO_2_, relative humidity) and culture media (DMEM/F12 for PNT2, WM793, HT29, A549; DMEM Low Glucose for DU-145; DMEM High Glucose for A375, HaCaT, A549; MEM with NEAA for Caco-2), supplemented with 10% fetal bovine serum (FBS) and 1% antibiotic solution (10,000 U penicillin and 10 mg streptomycin/mL). All cell lines, cell culture media, and supplements were from Merck (Seelze, Germany).

Before the experiment, cells were seeded onto 96-well plates for 24 h, at a density of 1.5 × 10^4^ cells/ well. Then the tested compounds (5–100 μg/mL) were added and incubated for 24 and 48 h. Cell viability was measured with the lactate dehydrogenase (LDH) assay by Clontech (Clontech, Mountain View, CA, USA), as described previously [49]. The absorbance was measured at 490 nm using a Biotek Synergy microplate reader (BioTek Instruments Inc., Winooski, VT, USA). Cell viability was expressed as a percentage of dead cells. Each experiment was conducted in triplicate. Doxorubicin was used as a reference cytostatic.

### 3.8. Data Analysis

The results were expressed as mean (±SD). Data were analyzed using Statistica v.13.3 (StatSoft, Tulsa, OK, USA). The IC_50_ values were calculated using OriginPro 2020b (64-bit) 9.7.5.184 (Academic) (OriginLab Corporation, Northampton, MA, USA). One-way analysis of variance (ANOVA) and the post-hoc Tukey multiple comparison test were used. The statistical significance was defined at the probability level of *p* < 0.05. Principal component analysis (PCA) and hierarchical cluster analysis (HCA) were performed using Statistica v.13.3 (StatSoft, Tulsa, OK, USA). Prior to PCA, the Bartlett’s sphericity test was conducted, and the Kaiser-Meyer-Olkin Measure of Sampling Adequacy index (KMO) was examined. In the simple model, for each compound tested (**1–4**), the cell lines were the cases, and the different concentrations of the compound were the parameters. This was followed by a PCA model in which the cell lines were the parameters, and the compounds (at different concentrations) were the cases. All PCA models were based on a correlation matrix. Hierarchical cluster analysis (CA) based on standardized data was performed using Ward’s method and Euclidean distance. The results were grouped based on Mojena’s rule.

Graphs were generated in Excel 365 (Microsoft Office) or Statistica v. 13.3 (StatSoft, Tulsa, OK, USA), illustrations were made using CorelDraw 2021.5. (Corel Corporation). Chemical structures of saponins were drawn using Signals ChemDraw v. 23.1.2.7 (Revvity Signals Software, Inc., Waltham, MA, USA).

## 4. Conclusions

In conclusion, two hederagenin glucosides, including the novel compound 3-*O*-β-D-glucopyranosyl(1→3)-β-D-glucopyranosyl] hederagenin, were isolated for the first time from the fruits of *Oxybasis rubra* (L.) S.Fuentes, Uotila & Borsh. To the best of our knowledge, this is the first report on hederagenin-type saponins in this plant species, contributing to the broader understanding of the phytochemical profile of the Amaranthaceae. The results obtained from cytotoxicity screening across a broad panel of human cancer and normal cell lines of both isolates along with two commercially obtained closely related compounds, 28-*O*-β-D-glucopyranosyl hederagenin ester and free hederagenin, provide a rationale for further investigation of the saponins. Future studies should include mechanism-based assays, which were beyond the scope of the current work and represent a limitation, as well as in vivo evaluations to confirm therapeutic potential. Our results have revealed that a monodesmoside with a sugar chain linked at the C-3 position generally showed significantly higher cytotoxicity, which is consistent with observations by other authors. In the case of hederagenin derivatives tested, this effect depends, however, not only on the structure of the compound but also on its concentration and the intrinsic sensitivity of individual cell lines, which was evidenced by the results of a multivariate analysis of the cytotoxicity data. Additionally, the results of the current study offer some scientific support for the folk use of *Oxybasis rubra* in tumor-related conditions. Given its wide geographic distribution and the promising activity of its saponins, this species represents a valuable source of bioactive compounds for further pharmacological exploration.

## Figures and Tables

**Table 1:** ^13^C (125 MHz) and ^1^H (500 MHz) NMR spectroscopic data (pyridine-*d*_5_) of compound **1** (*J* in Hz).

No.	δ_C_	δ_H_ (*J* in Hz) *
1	38.48	0.93, 1.40
2	25.81	1.86, 2.18
3	81.76	4.22 dd (12.3, 4.4)
4	43.30	-
5	47.28	1.62
6	17.97	1.29, 1.66
7	32.69	1.22, 1.55
8	39.57	-
9	47.94	1.69
10	36.71	-
11	23.65	1.85, 2.00
12	122.29	5.40, t (3.5)
13	144.78	-
14	41.98	-
15	28.17	1.08, 2.10
16	23.51	1.86, 2.01
17	46.49	-
18	41.81	3.24, dd (13.7, 4.0)
19	46.27	1.22, 1.71
20	30.78	-
21	34.04	1.13, 1.37
22	33.09	1.75, 1.97
23	64.04	3.66, d (10.5), 4.26, d (7.5)
24	13.53	0.89, s
25	15.88	0.84, s
26	17.32	0.95, s
27	26.01	1.19, s
28	180.37	-
29	23.61	0.93, s
30	33.09	0.86, s
-OCH_3_		
3-*O*-β-D-Glc		
1	105.33	5.02, d (7.0)
2	74.27	3.99
3	88.65	4.00
4	69.44	4.06
5	77.73	3.75
6	62.18	4.34, 4.23
*O*-β-D-Glc’		
1	105.79	5.17, d (7.5)
2	75.35	3.99
3	78.08	4.19
4	71.37	4.12
5	78.56	3.97
6	62.26	4.48, 4.24

* Overlapping signals are reported without designated multiplicity.

**Table 2:** Cytotoxic activity of the tested compounds expressed as IC_50_ values [µg/mL].

			1	2	3	4	DOX 24 h
skin panel	**HTB-140**	24 h	26.23 ± 0.97 ^a^	>100	>100	>100	4.91 ± 0.15 ^b^
		48 h	18.19 ± 0.58 ^a^	>100	98.24 ± 6.46 ^b^	>100	-
	**A375**	24 h	18.00 ± 0.51 ^a^	32.53 ± 0.25 ^b^	46.67 ± 0.23 ^c^	>100 ^c^	0.43 ± 0.01
		48 h	12.73 ± 0.16 ^a^	30.10 ± 0.45 ^b^	36.81 ± 0.49 ^c^	>100	-
	**WM793**	24 h	27.57 ± 0.71 ^a^	13.82 ± 0.17 ^b^	>100	>100	>40
		48 h	17.16 ± 1.42 ^a^	6.52 ± 0.17 ^b^	>100	>100	-
	**HaCaT**	24 h	40.42 ± 1.99 ^a^	44.90 ± 0.76 ^b^	58.10 ± 2.04 ^c^	>100	4.21 ± 0.27 ^d^
		48 h	26.99 ± 0.70 ^a^	39.93 ± 0.52 ^b^	43.29 ± 1.47 ^c^	>100	-
prostate panel	**DU-145**	24 h	16.49 ± 0.75 ^a^	31.98 ± 1.61 ^b^	36.07 ± 0.17 ^c^	51.47 ± 2.06 ^d^	2.46 ± 0.11 ^e^
		48 h	11.69 ± 0.50 ^a^	21.09 ± 0.32 ^b^	27.04 ± 1.11 ^c^	20.28 ± 1.16 ^d^	-
	**PC3**	24 h	58.57 ± 2.39	>100	>100	>100	>40
		48 h	35.17 ± 0.32 ^a^	67.00 ± 0.96 ^b^	74.76 ± 5.81 ^c^	77.73 ± 3.77 ^c^	-
	**PNT2**	24 h	40.71 ± 1.24 ^a^	>100	53.84 ± 3.39 ^b^	91.96 ± 6.19 ^c^	1.09 ± 0.05 ^d^
		48 h	34.08 ± 0.79 ^a^	>100	37.76 ± 1.03 ^b^	75.14 ± 6.48 ^c^	-
thyroid panel	**FTC133**	24 h	24.52 ± 1.15 ^a^	41.10 ± 1.29 ^b^	>100	>100	4.65 ± 0.22 ^c^
		48 h	13.60 ± 0.85 ^a^	22.54 ± 1.13 ^b^	>100	>100	-
	**8505C**	24 h	35.89 ± 1.02	>100	>100	>100	>40
		48 h	25.77 ± 0.87	>100	>100	>100	-
gastrointestinal panel	**Caco-2**	24 h	21.61 ± 0.68 ^a^	>100	27.04 ± 0.35 ^b^	90.13 ± 3.63 ^c^	2.95 ± 0.22 ^d^
		48 h	19.27 ± 0.53 ^a^	36.81 ± 0.43 ^b^	24.16 ± 0.33 ^a^	74.89 ± 4.09 ^c^	-
	**HT29**	24 h	28.45 ± 0.88 ^a^	>100	43.97 ± 1.14 ^b^	>100	1.12 ± 0.08 ^c^
		48 h	24.96 ± 1.51 ^a^	63.80 ± 0.56 ^b^	38.12 ± 0.59 ^c^	90.33 ± 3.09 ^d^	-
	**HepG2**	24 h	13.21 ± 0.23 ^a^	94.01 ± 7.13 ^b^	80.51 ± 6.09 ^c^	>100	1.11 ± 0.06 ^d^
		48 h	10.84 ± 0.22 ^a^	60.48 ± 1.71 ^b^	68.00 ± 3.45 ^c^	>100	-
lung panel	**A549**	24 h	14.88 ± 0.88 ^a^	13.20 ± 0.67 ^b^	95.32 ± 3.93 ^c^	>100	1.09 ± 0.06 ^d^
		48 h	11.80 ± 0.54 ^a^	8.23 ± 0.29 ^a^	46.45 ± 2.12 ^b^	98.15 ± 10.66	--

Abbreviations and symbols: **1**: 3-*O*-β-D-glucopyranosyl(1→3)-β-D-glucopyranosyl] hederagenin, **2**: 3-*O*-β-D-glucopyranosyl(1→3)-β-D-glucopyranosyl] hederagenin 28-*O*-β-D-glucopyranosyl ester, **3**: 28-*O*-β-D-glucopyranosyl hederagenin ester, **4**: hederagenin, DOX: doxorubicin, HTB-140: malignant melanoma, A375: malignant melanoma, WM793: primary melanoma, HaCaT: normal keratinocytes, DU-145: metastatic prostate carcinoma, PC3: metastatic prostate carcinoma, PNT2: prostate epithelial cells, FTC133: follicular thyroid carcinoma, 8505C: undifferentiated thyroid carcinoma, Caco-2: colon adenocarcinoma, HT29: colon adenocarcinoma, HepG2: hepatocellular carcinoma, A549: lung carcinoma. Means in the same row that do not share the letter are significantly different. Notifications IC_50_ > 100, IC_50_ > 40 indicate the values could not have been determined.

**Table 3:** Basic features of the PCA model (cell lines as parameters).

Eigenvalues	% Total Variance (Cumulative (%))	Parameters	PC1 Factor Loadings	PC2 Factor Loadings
10.52	80.93%	HTB-140	−0.922834	−0.249766
1.53	11.79%	A375	−0.962276	0.213910
	(92.72%)	WM793	−0.743319	0.655977
		HaCaT	−0.914098	0.229255
		DU-145	−0.968428	0.044078
		PC3	−0.908558	−0.220620
		PNT2	−0.894369	−0.350598
		FTC133	−0.902028	0.366692
		8505C	−0.888196	−0.156901
		Caco-2	−0.858857	−0.468982
		HT29	−0.917001	−0.367546
		HepG2	−0.938572	−0.082547
		A549	−0.854339	0.497125

## Data Availability

Data is contained within the article or Supplementary Materials.

## Supplementary Materials

The following supporting information can be downloaded at: https://www.mdpi.com/article/10.3390/molecules30153126/s1, Figure S1. ^13^C NMR (125 MHz, pyridine-*d*_5_) spectrum of compound **1**. Figure S2. ^1^H NMR (500 MHz, pyridine-*d*_5_) spectrum of compound **1**. Figure S3. HSQC (pyridine-*d*_5_) spectrum of compound **1**. Figure S4. HMBC (pyridine-*d*_5_) spectrum of compound **1**. Figure S5. H2BC (pyridine-*d*_5_) spectrum of compound **1**. Figure S6. COSY (pyridine-*d*_5_) spectrum of compound **1**. Figure S7. TOCSY (pyridine-*d*_5_) spectrum of compound **1**. Figure S8. ROESY (pyridine-*d*_5_) spectrum of compound **1**. Figure S9. HPLC (ELSD) chromatogram of compound **1**. Figure S10. Calculated formula for compound **1** and HR-ESI-MS spectrum (positive-ion mode) of compound **1**. Figure S11. ESI-MS spectra (positive and negative ion mode) of compound **1**. Figure S12. ^1^H NMR (500 MHz, methanol-*d*_4_) spectrum of compound **2**. Figure S13. ^13^C (125 MHz, methanol-*d*_4_) spectrum of compound **2**. Figure S14. HSQC (methanol-*d*_4_) spectrum of compound **2**. Figure S15. HMBC (methanol-*d*_4_) spectrum of compound **2**. Figure S16. H2BC (methanol-*d*_4_) spectrum of compound **2**. Figure S17. COSY (methanol-*d*_4_) spectrum of compound **2**. Figure S18. TOCSY (methanol-*d*_4_) spectrum of compound **2**. Figure S19. ROESY (methanol-*d*_4_) spectrum of compound **2**. Figure S20. HPLC (ELSD) chromatogram of compound **2**. Figure S21. Calculated formula for compound **2** and HR-ESI-MS spectrum for compound **2**. Figure S22. ESI-MS spectrum (positive-ion mode) of compound **2**. Figure S23. TLC chromatogram of acid hydrolysis products of compounds **1** (1) and **2** (2) and sugar standards: arabinose (Ara), galactose (Gal); glucose (Glc); xylose (Xyl); rhamnose (Rha); glucuronic acid lactone (GlcA-lactone). Figure S24. TLC chromatogram of acid hydrolysis products of compounds **1** (1) and **2** (2) and triterpenes standards: hederagenin (HE), oleanolic acid (OA). Table S1. ^1^H (500 MHz) and ^13^C (125 MHz) NMR spectral data (δ ppm) for compound **2** (methanol-*d*_4_). Table S2. Cytotoxic activity of the tested compounds expressed as IC_50_ values [µM].

## Abbreviations

The following abbreviations are used in this manuscript:

CC	Column chromatography
CHCl_3_	Chloroform
COSY	Correlated spectroscopy
GI	Gastrointestinal tract
DOX	Doxorubicin
ESI	Electrospray ion
HCA	Hierarchical cluster analysis
HMBC	Heteronuclear multiple bond correlation
HPLC	High-performance column chromatography
HSQC	Heteronuclear single quantum coherence
IC_50_	Concentration with 50% of maximal inhibitory effect
KMO	Kaiser-Meyer-Olkin measure of sampling adequacy index
LC-MS	Liquid chromatography–mass spectrometry
LDH	Lactate dehydrogenase
MPLC	Medium-pressure liquid chromatography
NMR	Nuclear magnetic resonance
PCA	Principal component analysis
pTLC	Preparative thin-layer chromatography
ROESY	Rotating-frame Overhauser enhancement spectroscopy
TOCSY	Total correlation spectroscopy
