# Supplementary material for: Saponins from Oxybasis rubra (L.) S.Fuentes, Uotila & Borsh: Comparative Assessment of Cytotoxic Potential Against a Wide Panel of Cancer Cell Lines
Source: Molecules. 2025 Jul 25;30(15):3126. doi: 10.3390/molecules30153126 (PMC12348107; doi:10.3390/molecules30153126)
Supplement: Supplementary file 1 [file molecules-30-03126-s001.zip › molecules-3749753-supplementary.pdf]

Supplementary Materials

Title: Saponins from *Oxybasis rubra* (L.) S.Fuentes, Uotila & Borsh: comparative assessment of cytotoxic potential against a wide panel of cancer cell lines

Authors: Karolina Grabowska[1], Adam Mynarski[1], Agnieszka Galanty[1], Dagmara Wróbel-Biedrawa[1], Paweł Żmudzki[2,3] and Irma Podolak[1*]

1 Chair of Pharmacognosy, Jagiellonian University Medical College, 9 Medyczna Str., 30-688 Cracow, Poland; karolina1.grabowska@uj.edu.pl (K.G.); adammynar44@o2.pl (A.M.); agnieszka.galanty@uj.edu.pl (A.G.); dagmara.wrobel-biedrawa@uj.edu.pl (D.W.B); irma.podolak@uj.edu.pl (I.P.)

2 Department of Medicinal Chemistry, Jagiellonian University Medical College, 9 Medyczna Str., 30-688 Cracow, Poland; pawel.zmudzki@uj.edu.pl (P.Ż.)

3 Center for the Development of Therapies for Civilization and Age-Related Diseases, Jagiellonian University Medical College, Skawińska 8, 31-066 Krakow, Poland

Abstract

Two triterpene saponins, hederagenin glucosides, including a novel monodesmoside: 3-*O*-β-D-glucopyranosyl(1→3)-β-D-glucopyranosyl] hederagenin (compound **1**), were isolated from the fruits of *Oxybasis rubra* (L.) S.Fuentes, Uotila & Borsh (Amaranthaceae). These compounds, together with hederagenin itself (compound **4**) and a commercially available 28-*O*-β-D-glucopyranosyl hederagenin ester (compound **3**), were evaluated for cytotoxicity and selectivity across a wide panel of human cancer cell lines (skin, prostate, gastrointestinal, thyroid and lung). All four compounds exhibited dose- and time-dependent effects, with varying potency depending on the specific cancer type. The isolated bidesmosidic saponin (3-*O*-β-D-glucopyranosyl(1→3)-β-D-glucopyranosyl] hederagenin 28-*O*-β-D-glucopyranosyl ester – compound **2**) showed the strongest activity and selectivity, with an IC_{50} = 6.52 µg/mL after 48h incubation against WM793 melanoma, and almost no effect on normal HaCaT skin cells (IC_{50} =39.94 µg/mL). Multivariate analysis of the obtained data using principal component analysis (PCA) and hierarchical cluster analysis (HCA) supported the assumption that cytotoxicity is influenced by the type of compound, its concentration and the intrinsic sensitivity of the cell line. Structure-activity observations between closely related hederagenin derivatives are also briefly presented.

Keywords: triterpene saponins, *Oxybasis rubra*, isolation, cytotoxicity, structure-activity observations, multivariate analysis, Amaranthaceae

Table of contents:

Figure S1. ^{13}C NMR (125 MHz, pyridine-d_5) spectrum of compound **1**.

Figure S2. ^{1}H NMR (500 MHz, pyridine-d_5) spectrum of compound **1**.

Figure S3. HSQC (pyridine-d_5) spectrum of compound **1**.

Figure S4. HMBC (pyridine-d_5) spectrum of compound **1**.

Figure S5. H2BC (pyridine-d_5) spectrum of compound **1**.

Figure S6. COSY (pyridine-d_5) spectrum of compound **1**.

Figure S7. TOCSY (pyridine-d_5) spectrum of compound **1**.

Figure S8. ROESY (pyridine-d_5) spectrum of compound **1**.

Figure S9. HPLC (ELSD) chromatogram of compound **1**.

Figure S10. Calculated formula for compound 1 and HR-ESI-MS spectrum (positive ion mode) of compound **1**.

Figure S11. ESI-MS spectra (positive and negative ion mode) of compound **1**.

Figure S12.^{1}H NMR (500 MHz, methanol-d_4) spectrum of compound **2**.

Figure S13. ^{13}C (125 MHz, methanol-d_4) spectrum of compound **2**.

Figure S14. HSQC (methanol-d_4) spectrum of compound **2**.

Figure S15. HMBC (methanol-d_4) spectrum of compound **2**.

Figure S16. H2BC (methanol-d_4) spectrum of compound **2**.

Figure S17. COSY (methanol-d_4) spectrum of compound **2**.

Figure S18. TOCSY (methanol-d_4) spectrum of compound **2**.

Figure S19. ROESY (methanol-d_4) spectrum of compound **2**.

Figure S20. HPLC (ELSD) chromatogram of compound **2**.

Figure S21. Calculated formula for compound **2** and HR-ESI-MS spectrum for compound **2**.

Figure S22. ESI -MS spectrum (positive ion mode) of compound **2**.

Figure S23. TLC chromatogram of acid hydrolysis products of compounds **1** (1) and **2** (2) and sugar standards: arabinose (Ara), galactose (Gal); glucose (Glc); xylose (Xyl); rhamnose (Rha); glucuronic acid lactone (GlcA-lactone).

Figure S24. TLC chromatogram of acid hydrolysis products of compounds **1** (1) and **2** (2) and triterpenes standards: hederagenin (HE), oleanolic acid (OA).

Table S1. ^{1}H (500 MHz) and ^{13}C (125 MHz) NMR spectral data (δ ppm) for compound **2** (methanol-d_4).

Table S2. Cytotoxic activity of the tested compounds expressed as IC_{50} values [μM].

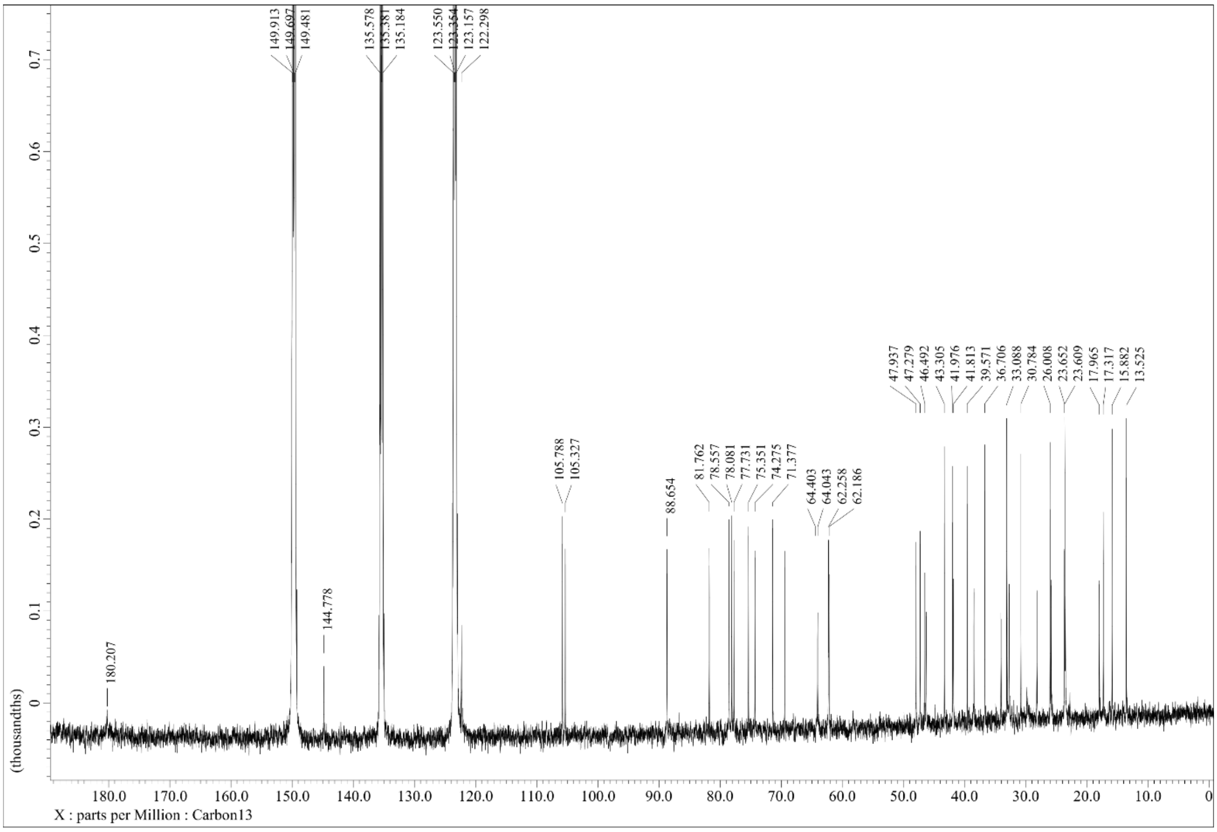

Figure S1. ^{13}C NMR (125 MHz, pyridine-d_5) spectrum of compound **1**.

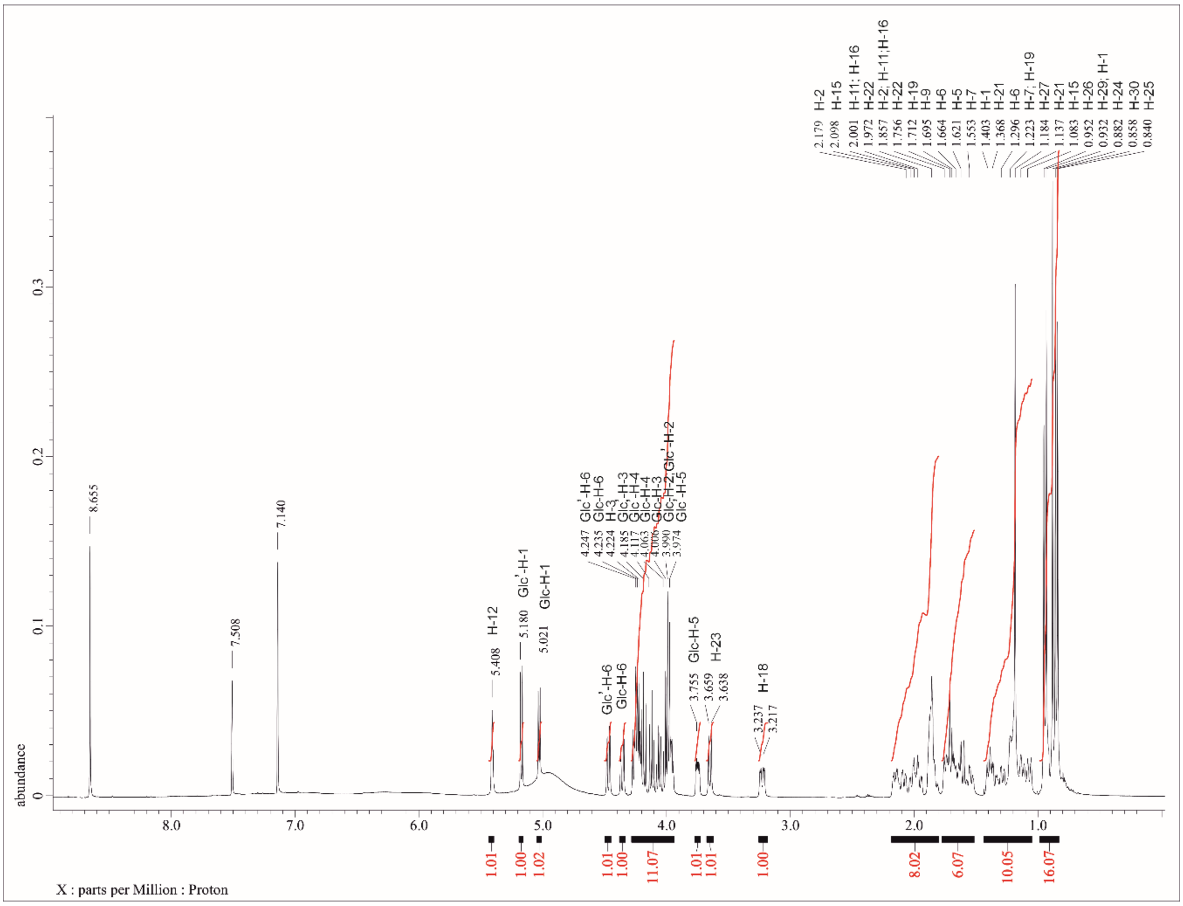

Figure S2.^{1}H NMR (500 MHz, pyridine-d_5) spectrum of compound **1**.

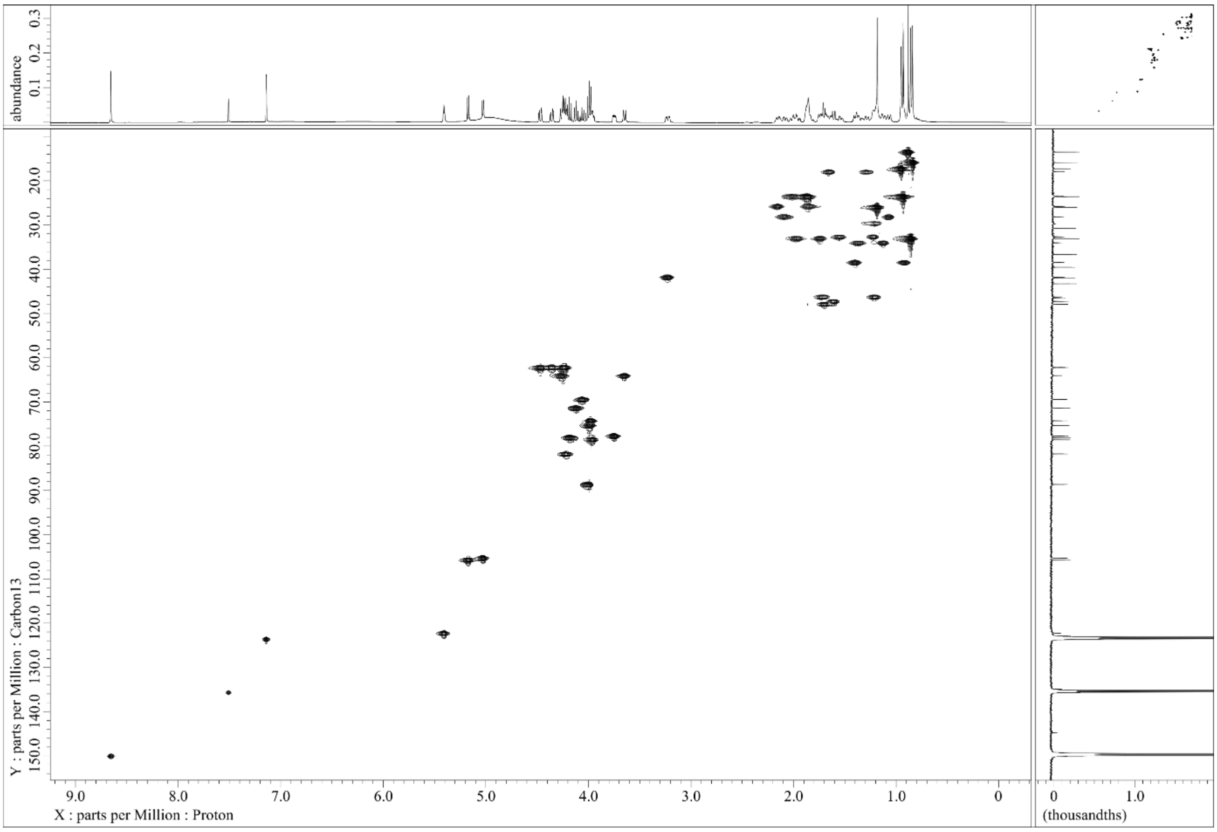

Figure S3. HSQC (pyridine-d_5) spectrum of compound **1**.

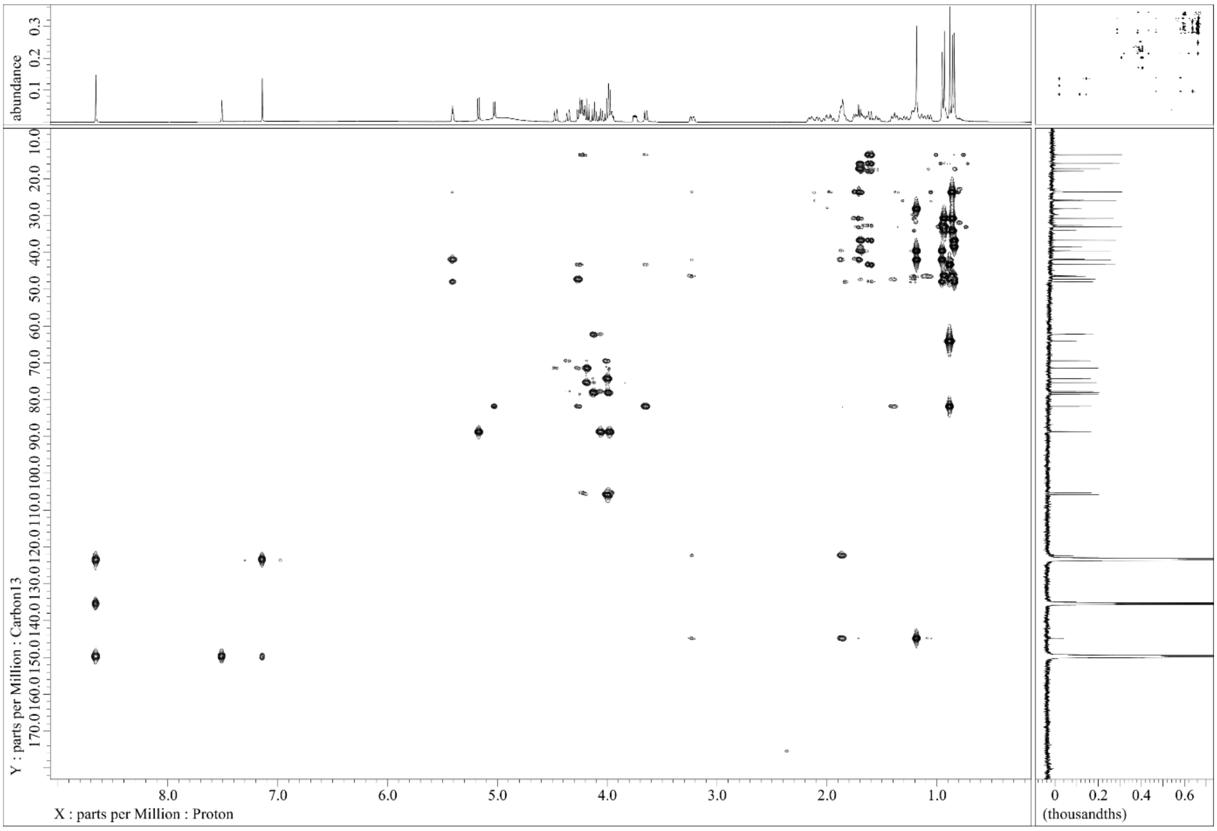

Figure S4. HMBC (pyridine-d_5) spectrum of compound **1**.

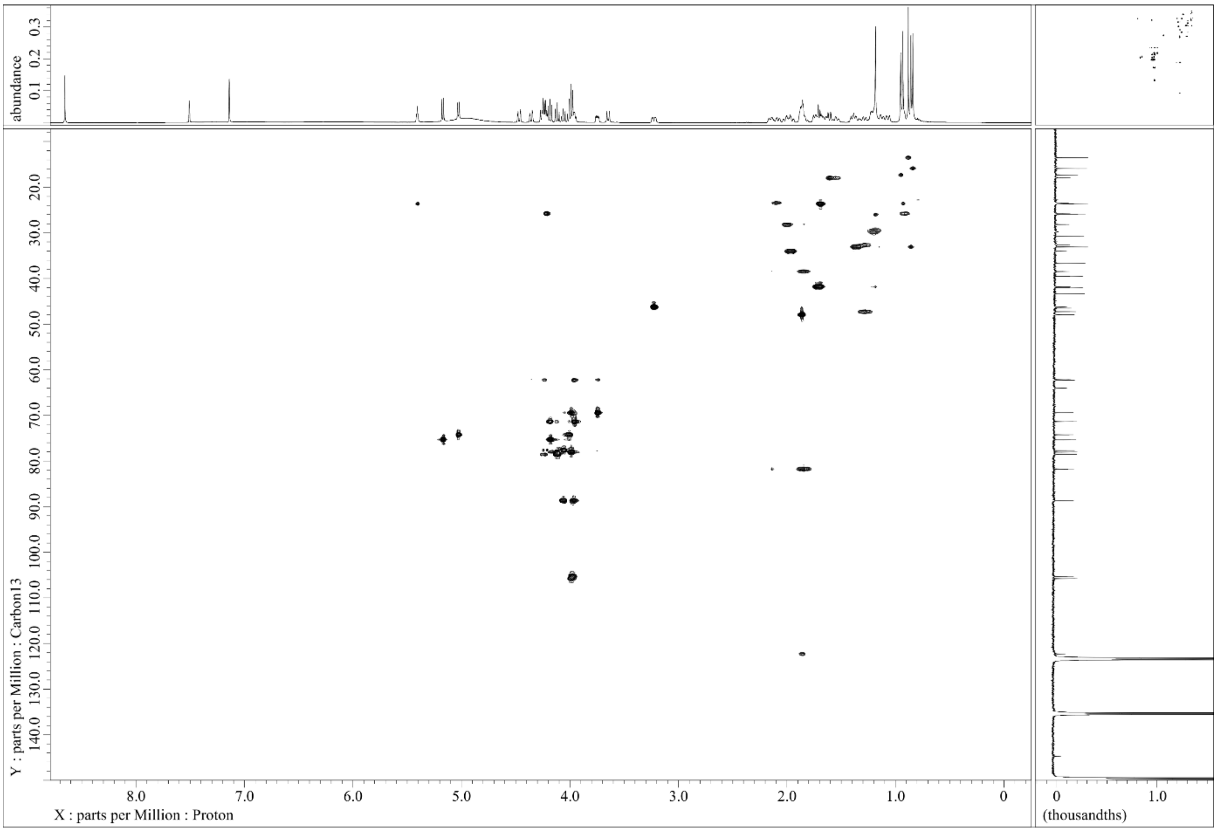

Figure S5. H2BC (pyridine-d_5) spectrum of compound **1**.

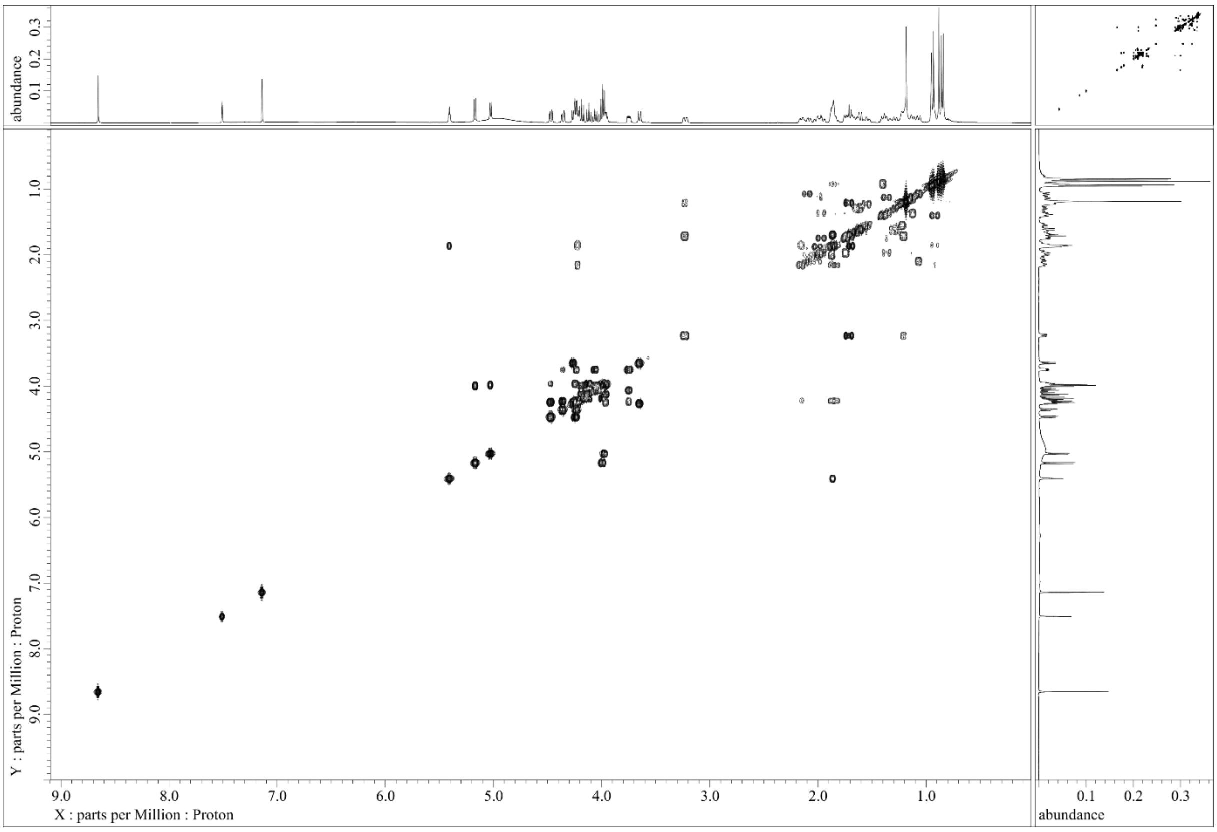

Figure S6. COSY (pyridine-d_5) spectrum of compound **1**.

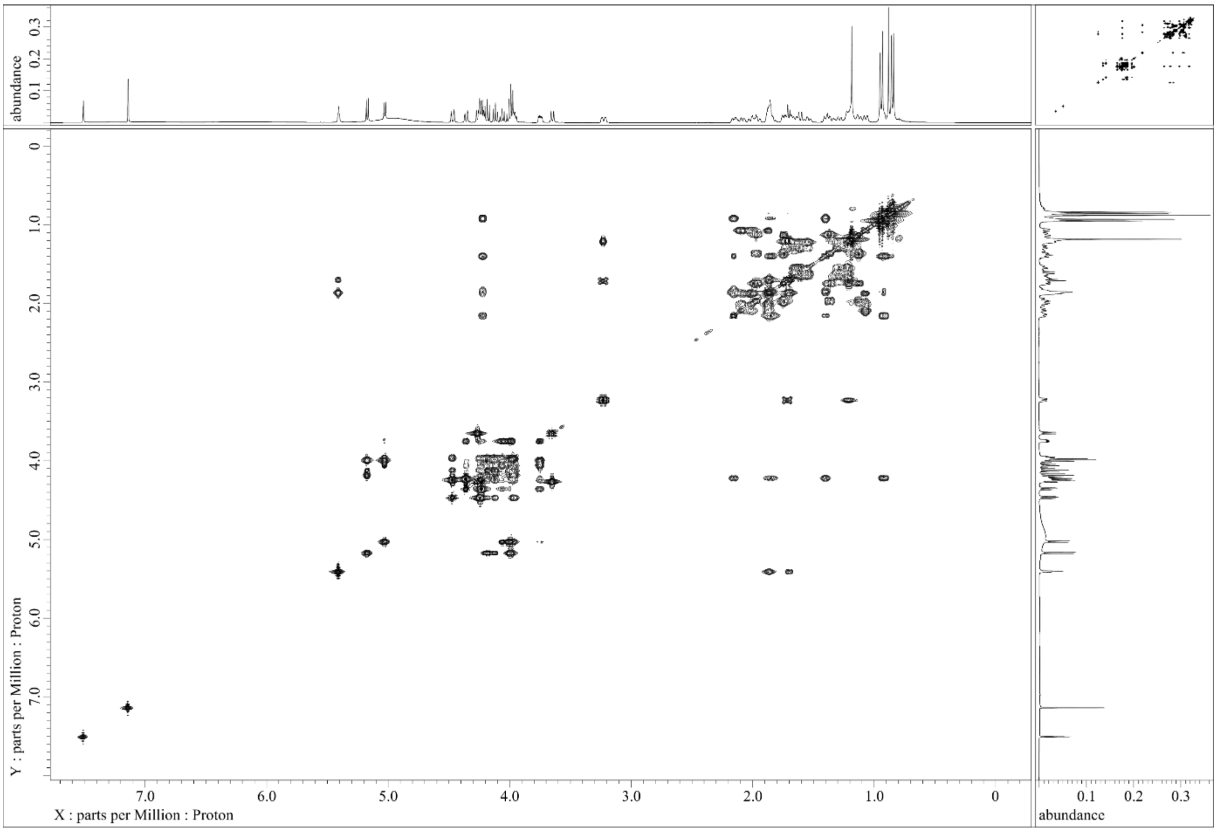

Figure S7. TOCSY (pyridine-d_5) spectrum of compound **1**.

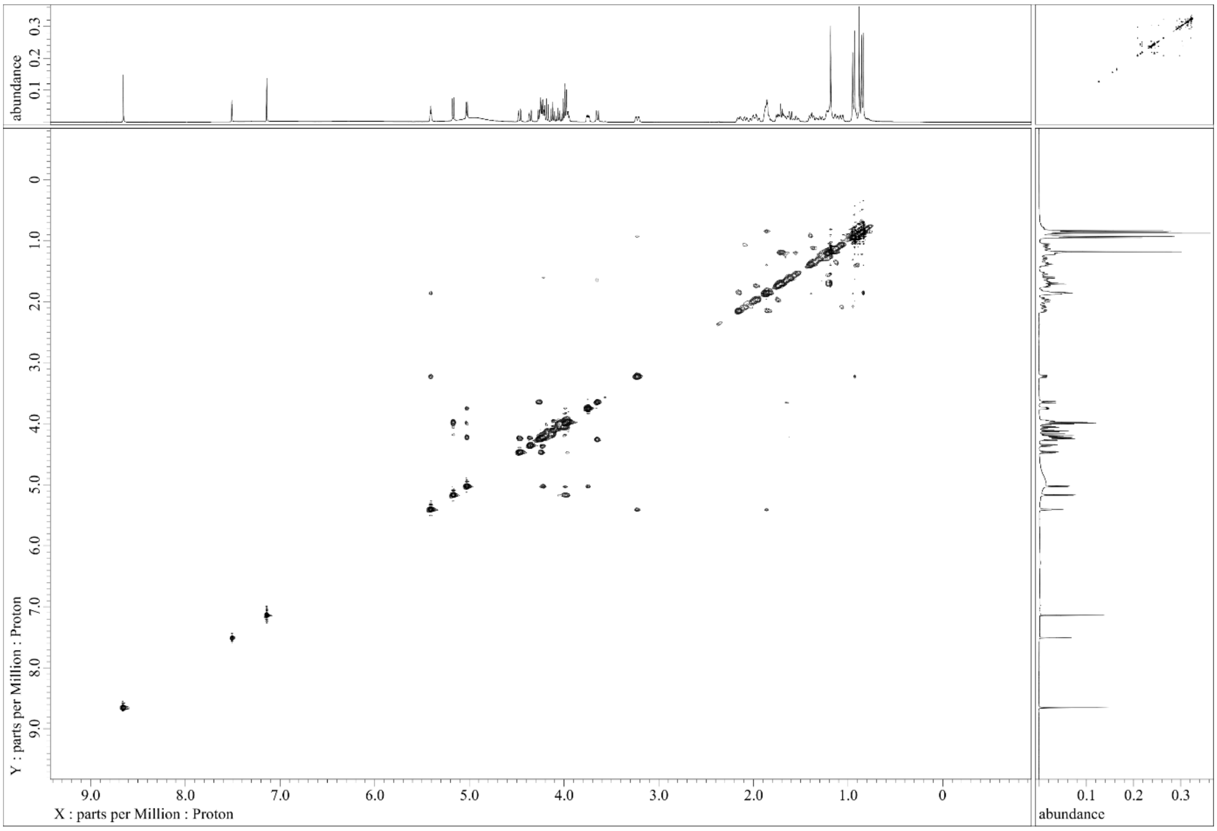

Figure S8. ROESY (pyridine-d_5) spectrum of compound **1**.

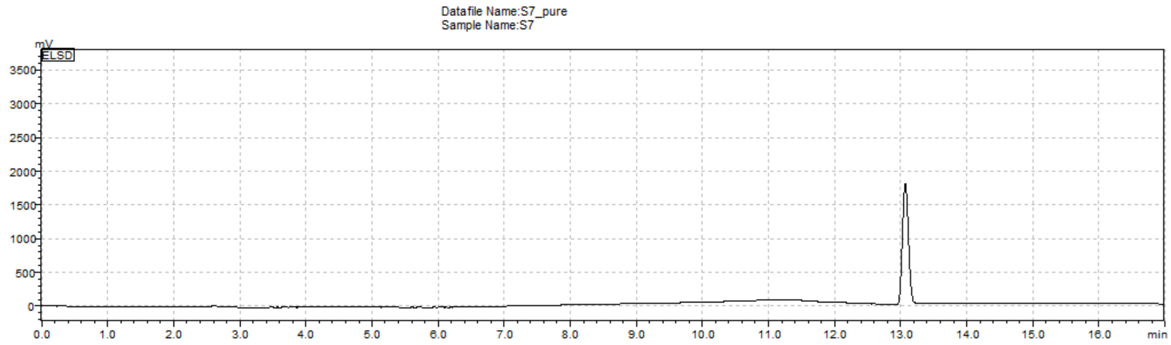

Figure S9. HPLC (ELSD) chromatogram of compound **1**.

Single Mass Analysis
Tolerance = 3.0 mDa / DBE: min = -1.5, max = 100.0
Element prediction: Off
Number of isotope peaks used for i-FIT = 3
Monoisotopic Mass, Even Electron Ions
136 formula(e) evaluated with 2 results within limits (all results (up to 1000) for each mass)
Elements Used:
C: 0-100 H: 0-200 O: 0-14 Na: 1-1

Mass	Calc. Mass	mDa	PPM	DBE	Formula	i-FIT	i-FIT Norm	Fit Conf %	C	H	O	Na
819.4519	819.4507	1.2	1.5	8.5	C42 H68 O14 Na	602.8	0.001	99.90	42	68	14	1
	819.4542	-2.3	-2.8	30.5	C60 H60 O Na	609.7	6.940	0.10	60	60	1	1

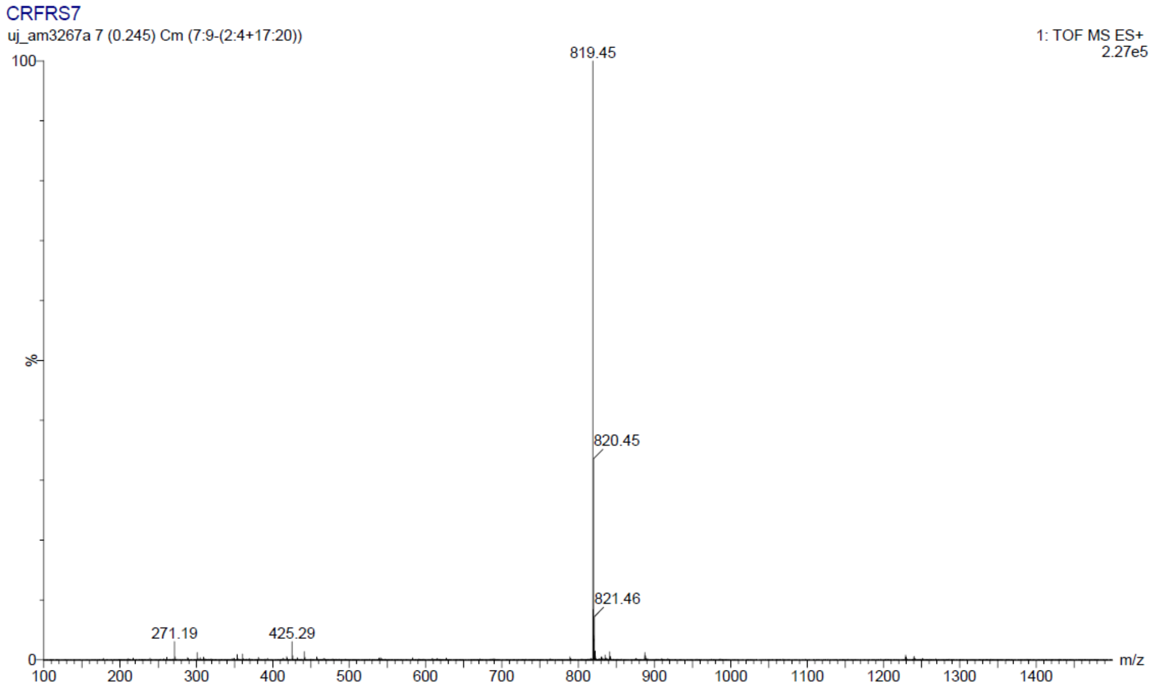

Figure S10. Calculated formula for compound **1** and HR-ESI-MS spectrum (positive ion mode) of compound **1**.

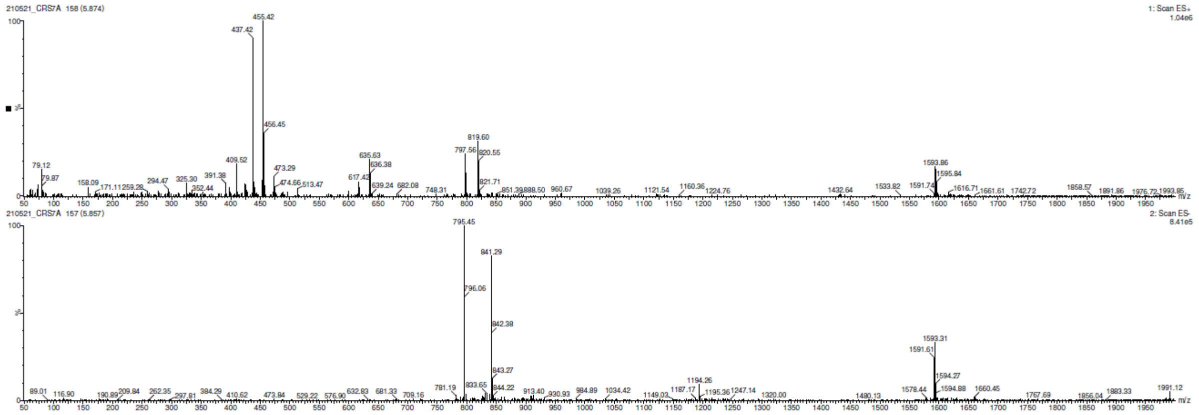

Figure S11. ESI-MS spectra (positive and negative ion mode) of compound **1**.

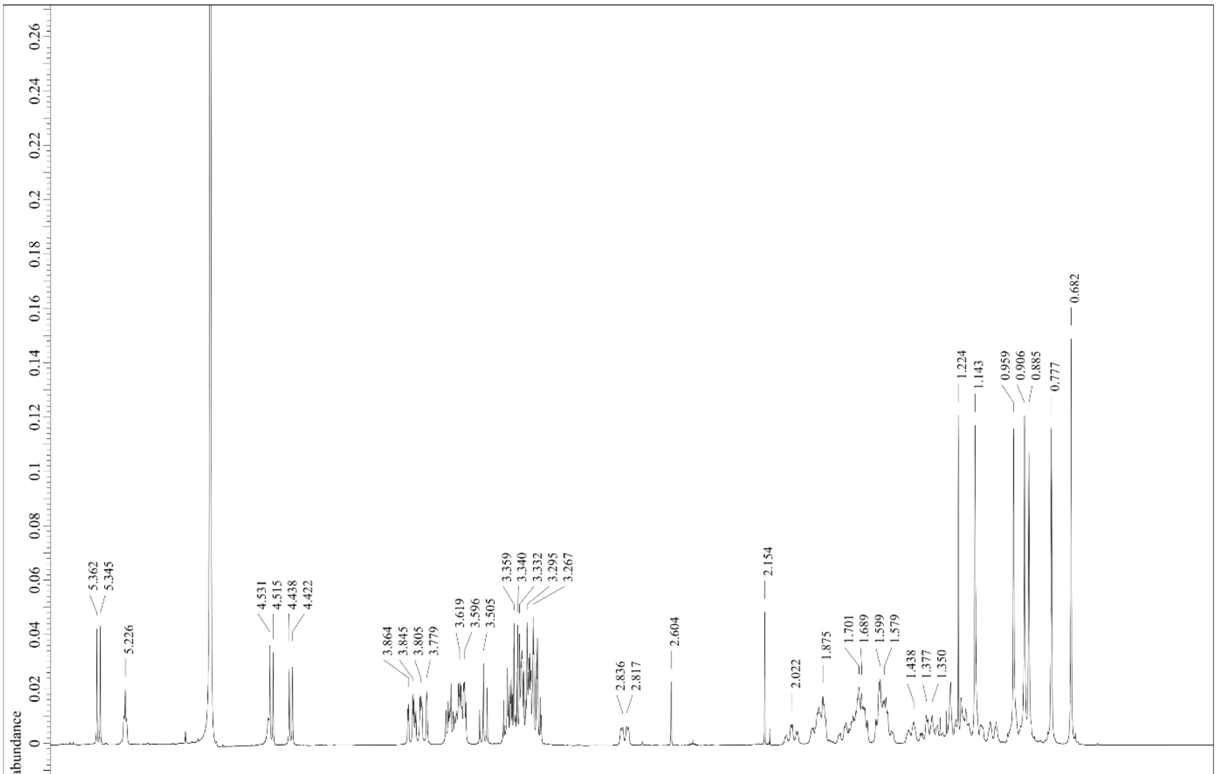

Figure S12.^{1}H NMR (500 MHz, methanol-d_4) spectrum of compound **2.**

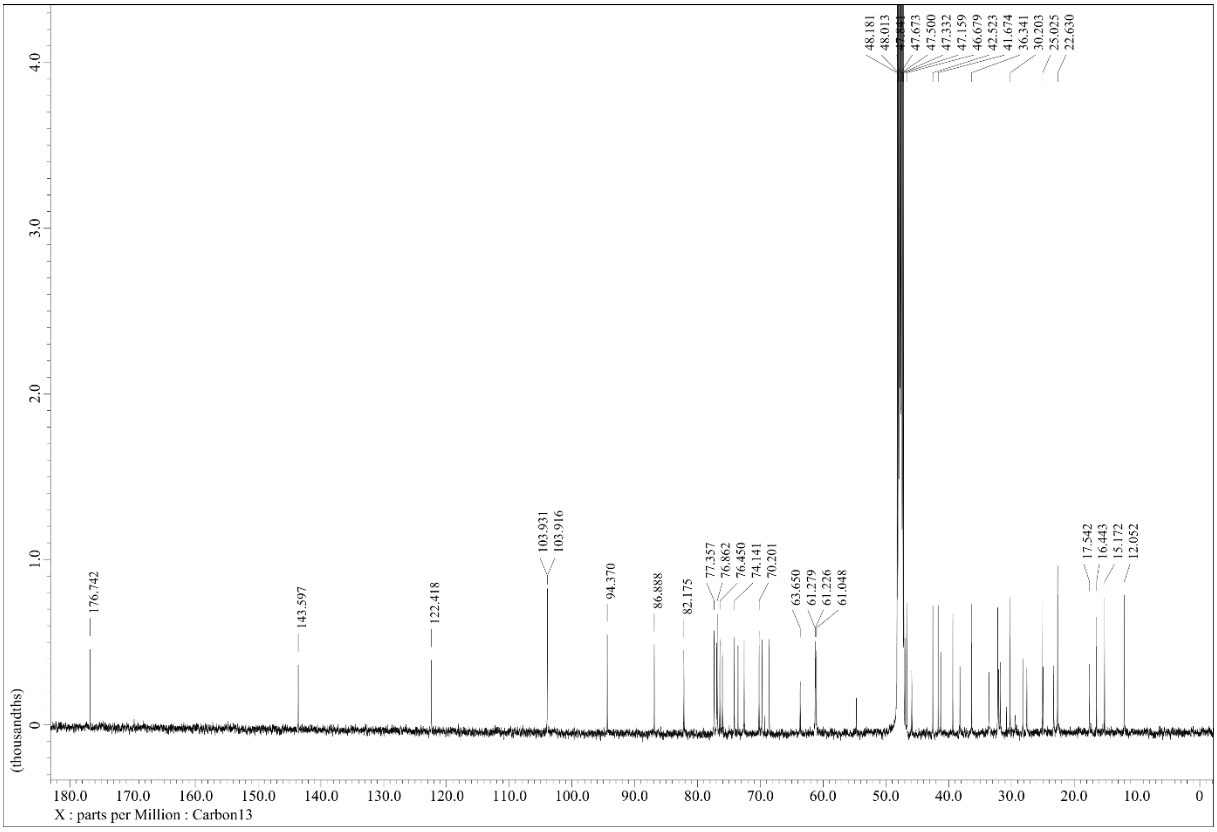

Figure S13. ^{13}C (125 MHz, methanol-d_4) spectrum of compound **2.**

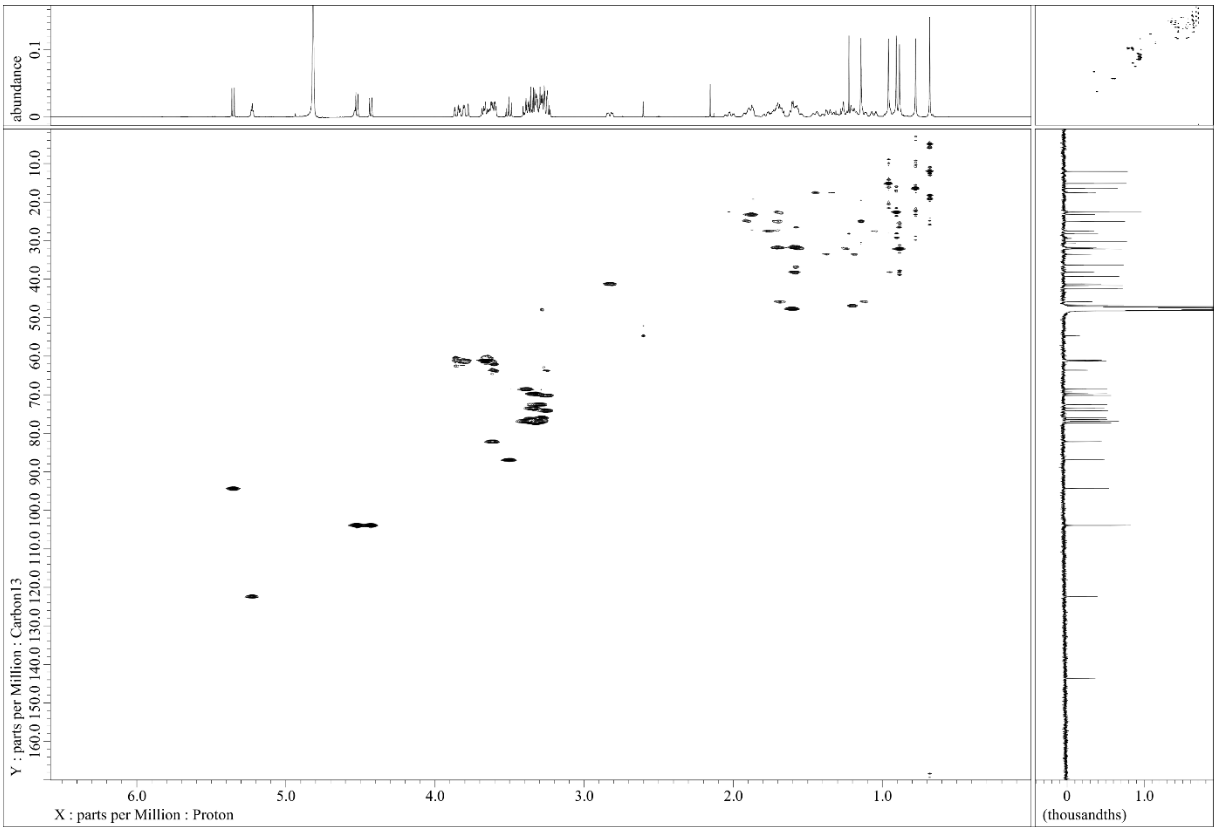

Figure S14. HSQC (methanol-d_4) spectrum of compound **2.**

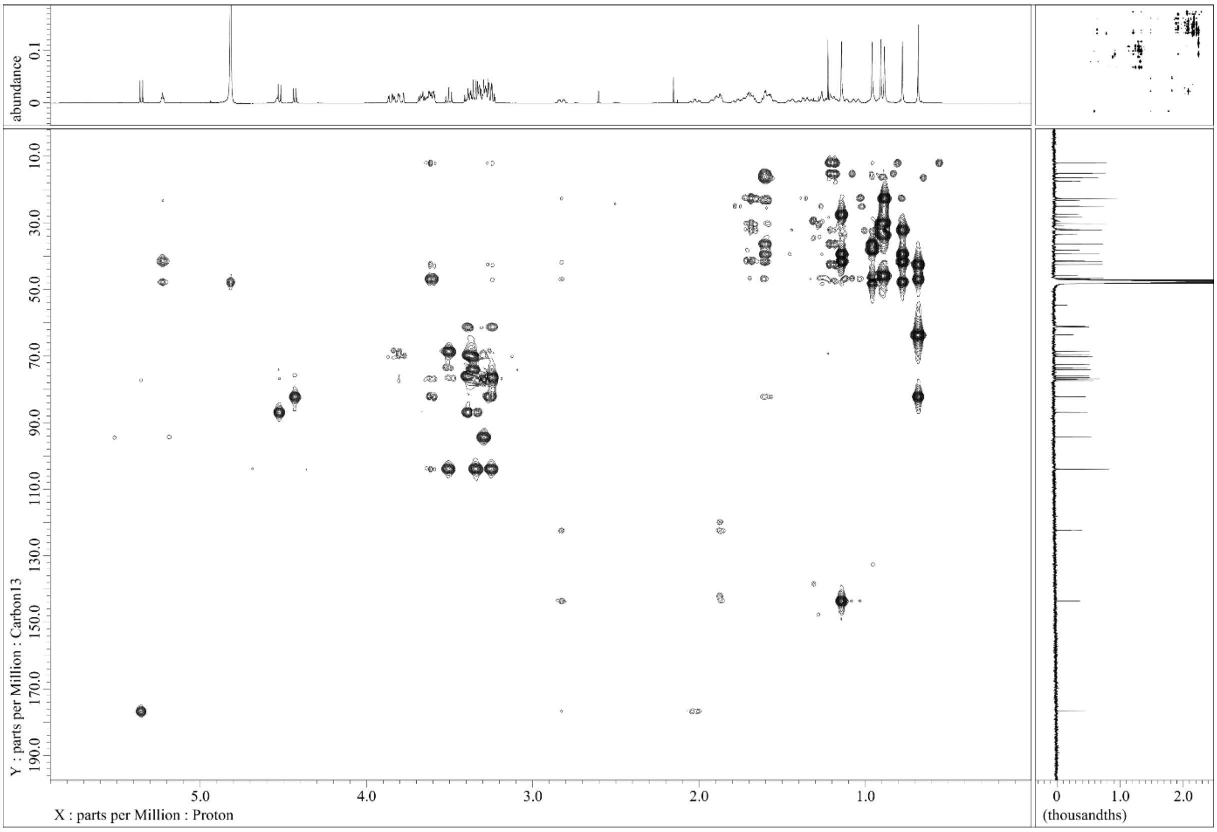

Figure S15. HMBC (methanol-d_4) spectrum of compound **2.**

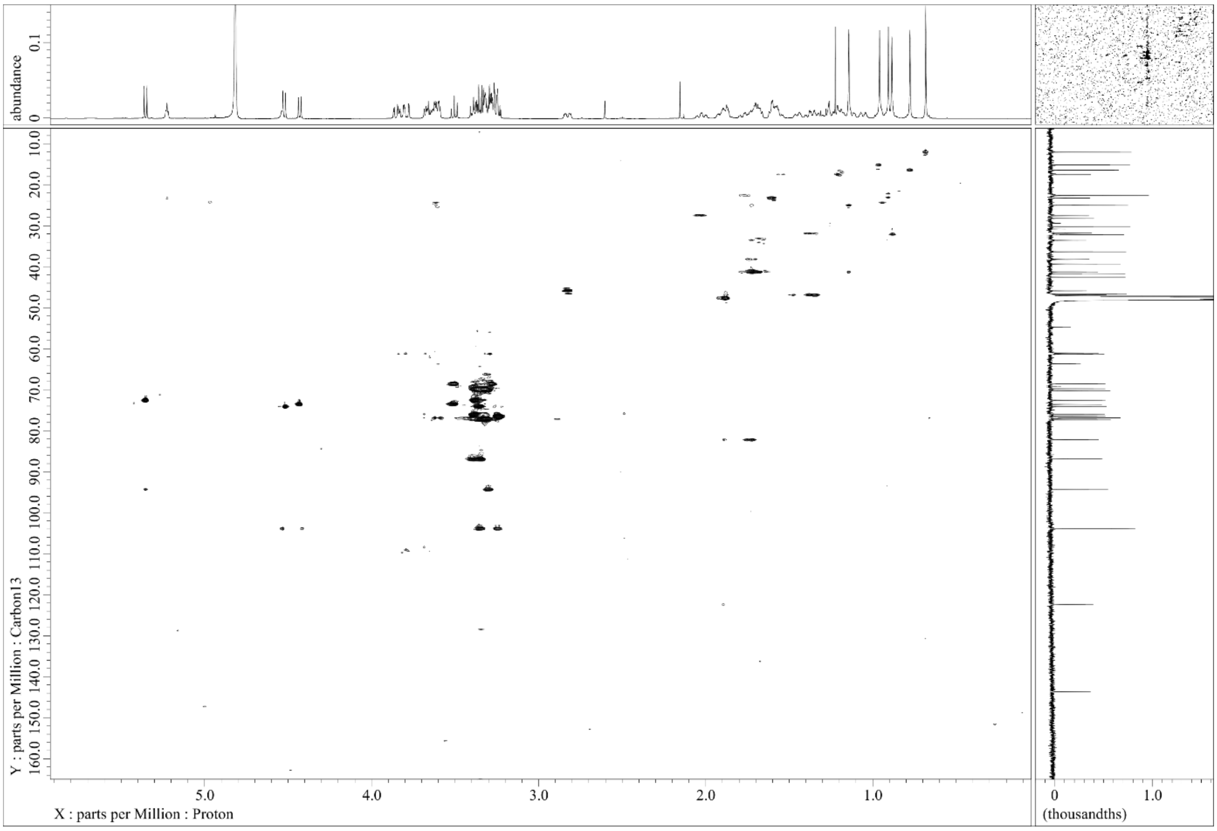

Figure S16. H2BC (methanol-d_4) spectrum of compound **2.**

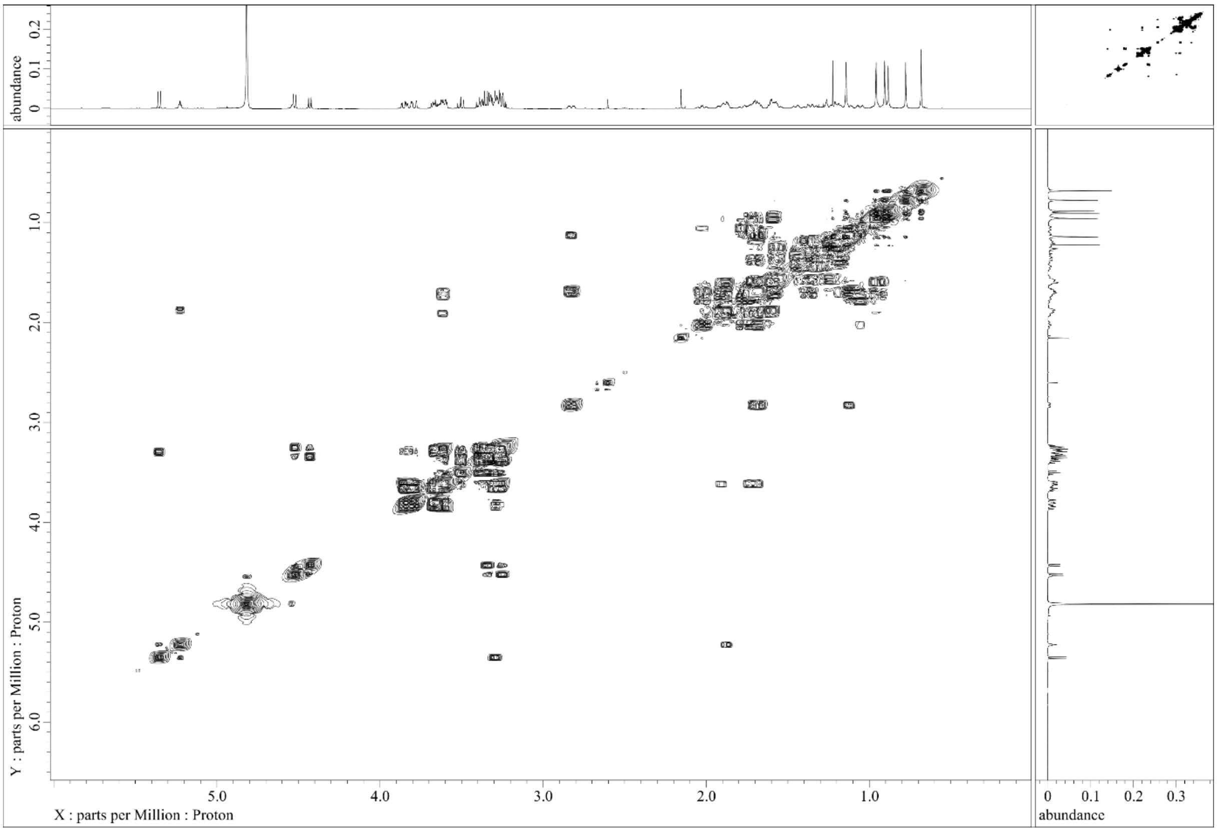

Figure S17. COSY (methanol-d_4) spectrum of compound **2**.

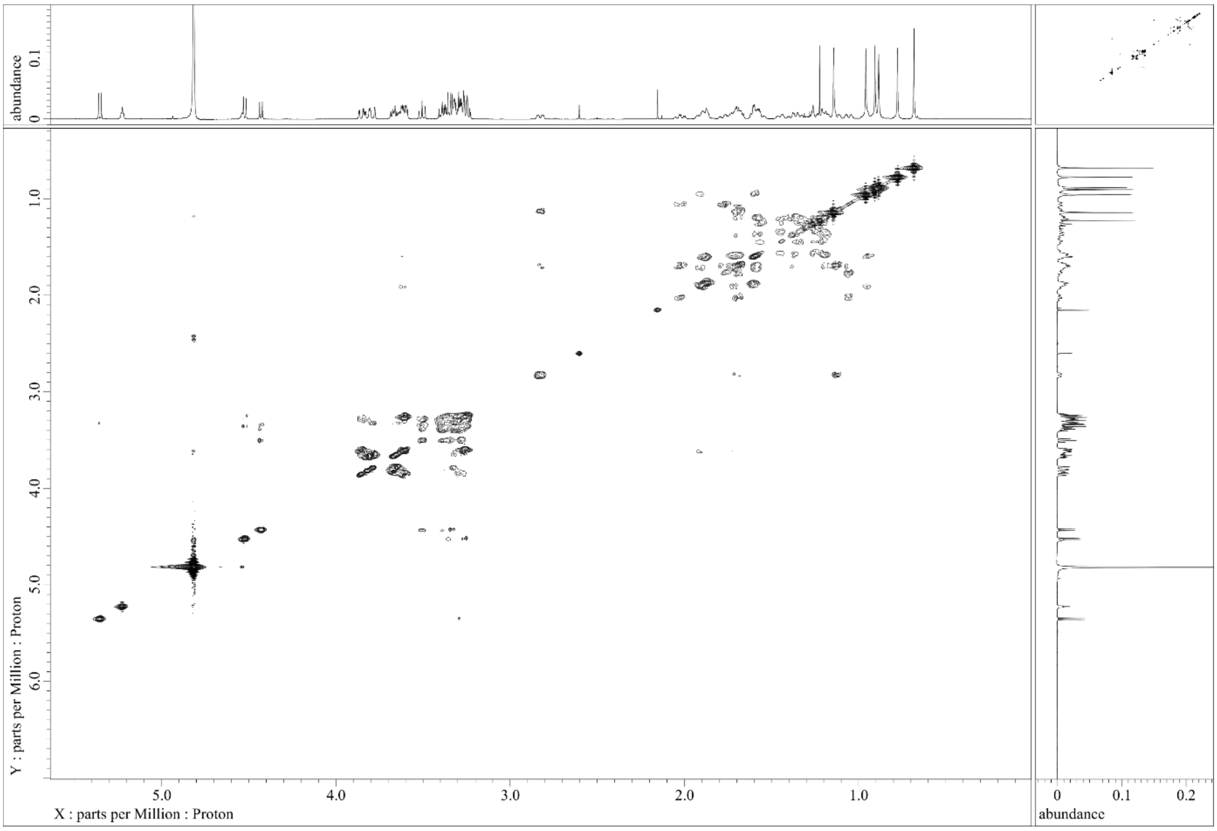

Figure S18. TOCSY (methanol-d_4) spectrum of compound **2.**

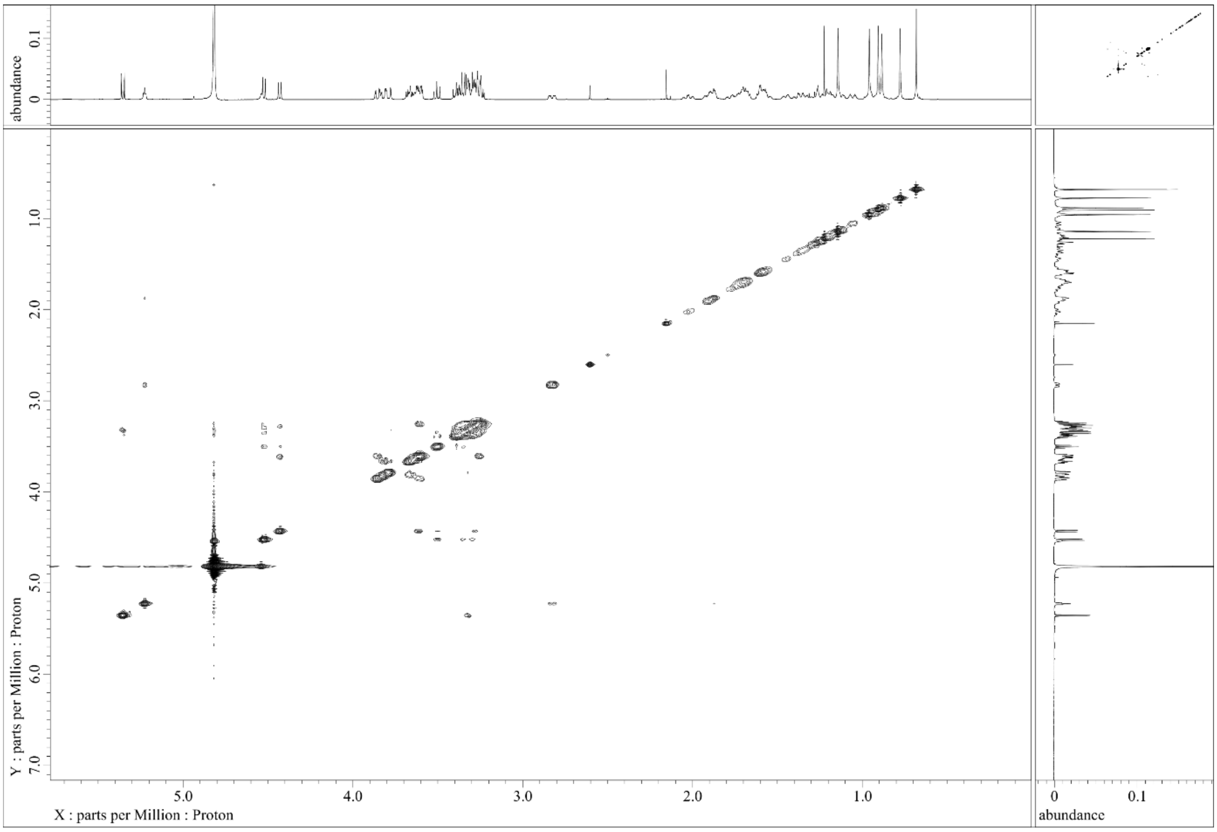

Figure S19. ROESY (methanol-d_4) spectrum of compound **2.**

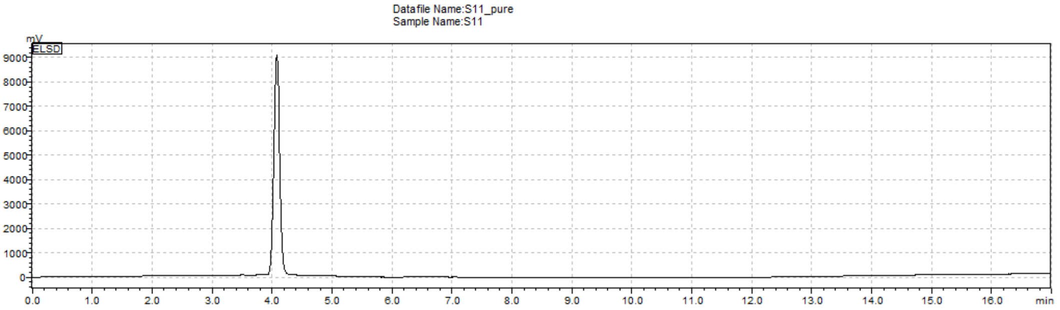

Figure S20. HPLC (ELSD) chromatogram of compound **2**

Elements Used:

C: 0-100 H: 0-200 O: 19-20 Na: 1-1

Mass	Calc. Mass	mDa	PPM	DBE	Formula	i-FIT	i-FIT Norm	Fit Conf %	C	H	O	Na
981.5032	981.5035	-0.3	-0.3	9.5	C48 H78 O19 Na	188.4	n/a	n/a	48	78	19	1

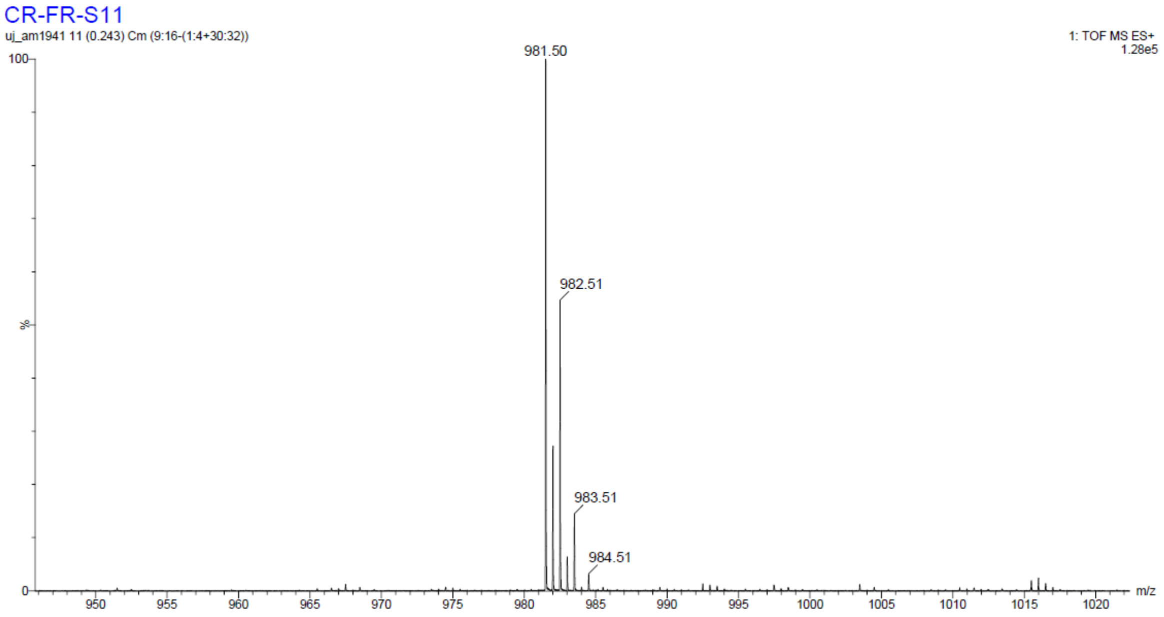

Figure S21. Calculated formula for compound **2** and HR-ESI-MS spectrum for compound **2.**

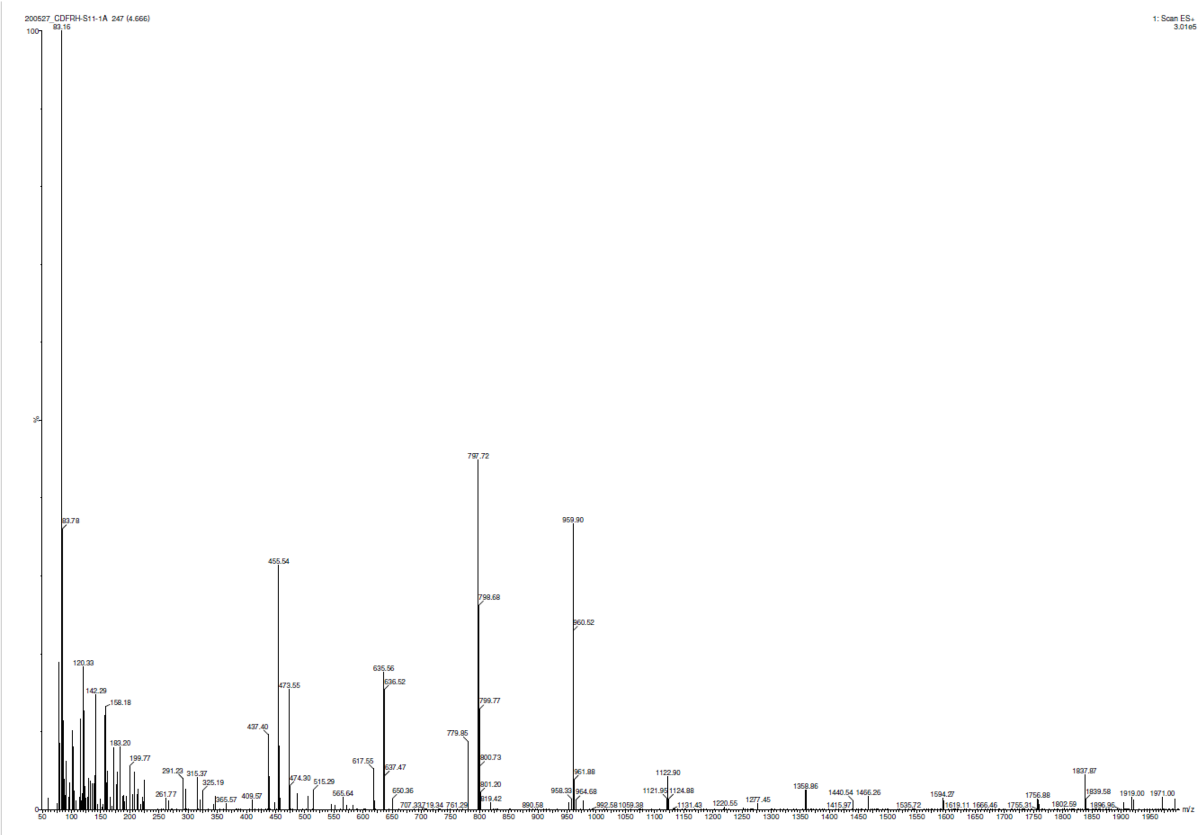

Figure S22. ESI -MS spectrum (positive ion mode) of compound **2**.

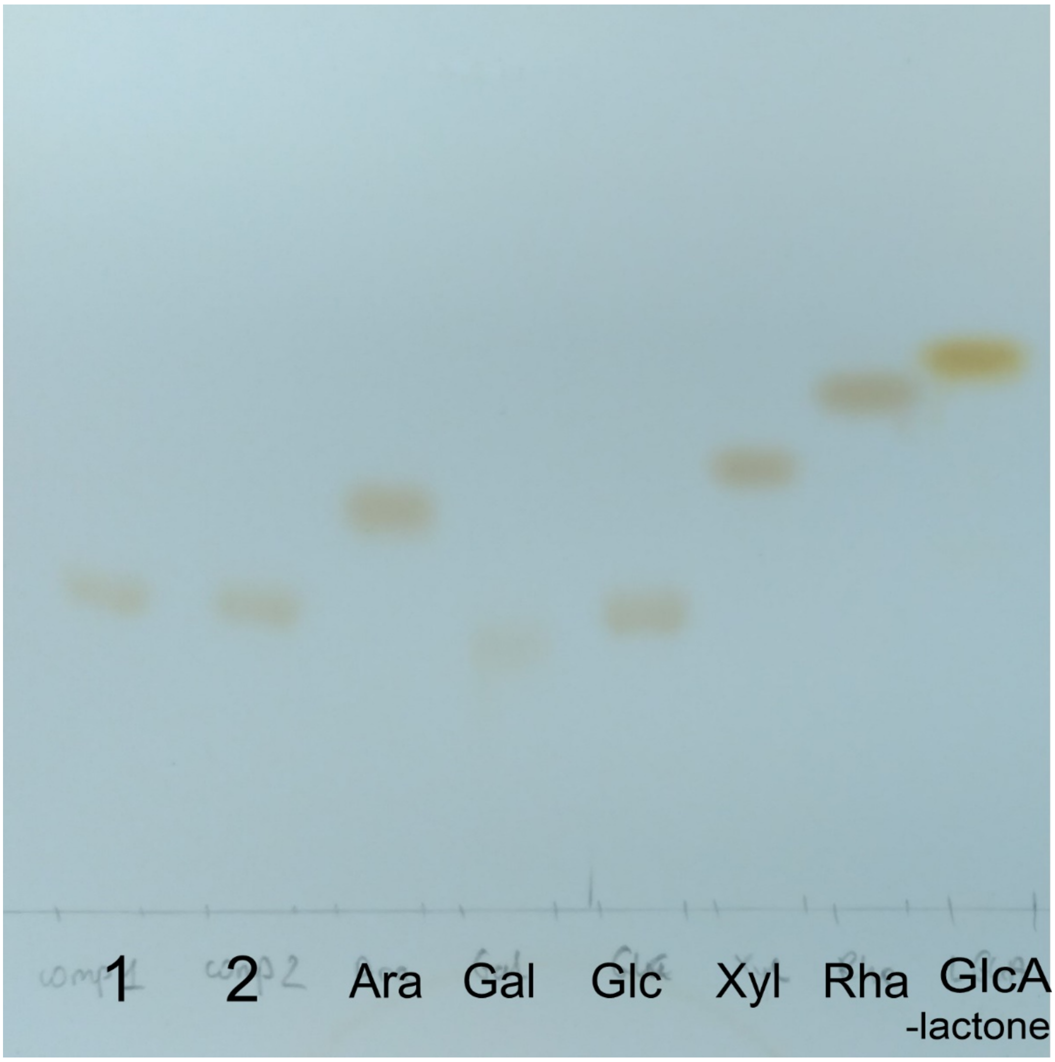

Figure S23. TLC chromatogram of acid hydrolysis products of compounds **1** (1) and **2** (2) and sugar standards: arabinose (Ara), galactose (Gal); glucose (Glc); xylose (Xyl); rhamnose (Rha); glucuronic acid lactone (GlcA-lactone).

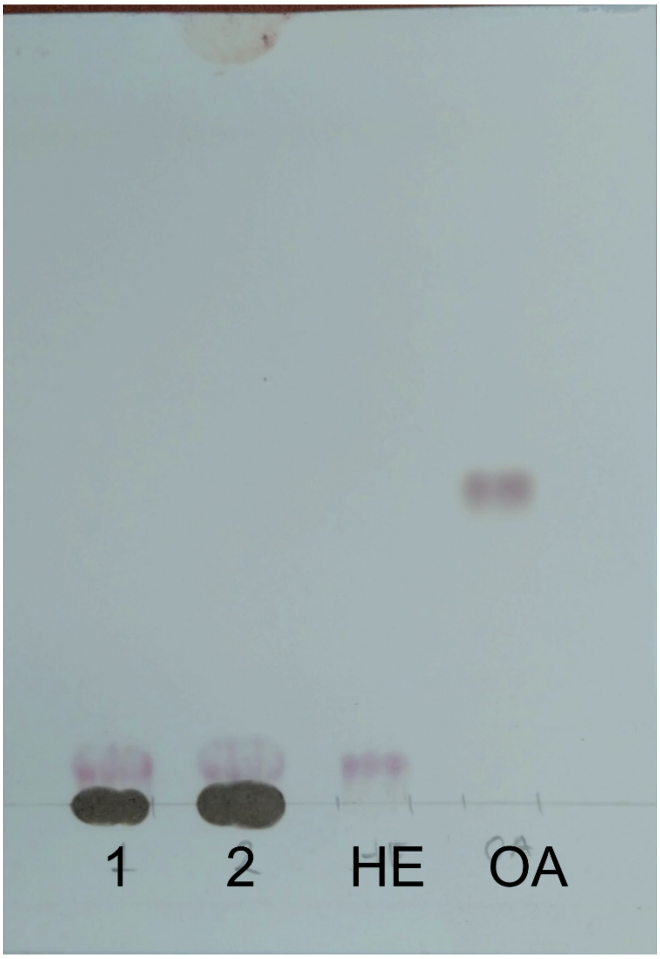

Figure S24. TLC chromatogram of acid hydrolysis products of compounds **1** (1) and **2** (2) and triterpenes standards: hederagenin (HE), oleanolic acid (OA).

Table S1. ^{13}C (125 MHz) and ^{1}H (500 MHz) NMR spectroscopic data (methanol-d_4) of compound **2** (*J* in Hz)

No.	δ_C	δ_H (*J* in Hz)*
1	38.20	0.95, 1.58
2	25.03	1.70, 1.91
3	82.18	3.61
4	42.52	-
5	46.88	1.21
6	17.54	1.33, 1.44
7	31.79	1.58 ,1.70
8	39.37	-
9	47.95	1.60
10	36.34	-
11	23.25	0.77, 1.87
12	122.42	5.22, t (3.5)
13	143.60	-
14	41.67	-
15	27.55	1.04, 1.77
16	22.63	1.70, 2.02
17	46.68	-
18	41.27	2.81
19	45.87	1.11, 1.69
20	30.20	-
21	33.56	1.19, 1.38
22	32.06	1.23, 1.57
23	63.65	3.25, 3.60
24	12.05	0.68
25	15.17	0.96
26	16.44	0.78
27	24.97	1.14
28	176.74	-
29	32.17	0.89
30	22.63	0.91
-OCH_3		
3-*O*-β-D-Glc		
1	103.93	4.43, d (7.7)
2	73.55	3.34
3	86.89	3.50
4	68.56	3.39
5	76.04	3.28
6	61.28	3.68, 3.80
β-D-Glc′		
1	103.92	4.53, d (8.0)
2	74.14	3.25
3	76.86	3.36
4	70.20	3.24
5	76.45	3.28
6	61.23	3.77, 3.86
28-*O*-β-D-Glc		
1	94.37	5.35 d (8.0)

2	72.56	3.30
3	76.96	3.37
4	69.75	3.33
5	77.36	3.33
6	61.05	3.65, 3.80

*Overlapping signals are reported without designated multiplicity

Table S2. Cytotoxic activity of the tested compounds expressed as IC_{50} values [μM]

			1	2	3	4	DOX 24h
skin panel	**HTB-140**	24h	32.95±1.21[a]	>100	>100	>100	9.03±0.28[b]
		48h	22.84±0.73[a]	>100	154.73±10.17[b]	>100	-
	**A375**	24h	22.61±0.65[a]	33.91±0.26[b]	73.50±0.36[c]	>100	0.79±0.01[d]
		48h	16.00±0.20[a]	31.38±0.47[b]	57.98±0.78[c]	>100	-
	**WM793**	24h	34.63±0.89[a]	14.40±0.17[b]	>100	>100	>100
		48h	21.55±1.78[a]	6.79±0.18[b]	>100	>100	-
	**HaCaT**	24h	50.77±2.50[a]	46.81±0.79[a]	91.51±3.21[b]	>100	7.75±0.50[c]
		48h	33.90±0.88[a]	41.63±0.54[b]	68.19±2.31[c]	>100	-
prostate panel	**DU-145**	24h	20.72±0.94[a]	33.34±1.68[b]	56.81±0.27[c]	108.87±4.35[d]	4.53±0.21[e]
		48h	14.68±0.63[a]	21.99±0.33[b]	42.59±1.75[c]	42.90±2.46[c]	-
	**PC3**	24h	73.57±3.00	>100	>100	>100	>100
		48h	44.18±0.40[a]	69.84±1.00[b]	117.74±9.14[c]	164.42±7.97[d]	-
	**PNT2**	24h	51.14±1.55[a]	>100	84.80±5.35[b]	194.51±13.09[c]	2.01±0.09[d]
		48h	42.81±0.99[a]	>100	59.47±1.63[b]	158.92±13.70[c]	-
thyroid panel	**FTC133**	24h	30.80±1.44[a]	42.85±1.35[b]	>100	>100	8.56±1.35[c]
		48h	17.08±1.06[a]	23.50±1.18[b]	>100	>100	-
	**8505C**	24h	45.09±1.28	>100	>100	>100	>100
		48h	32.37±1.09	>100	>100	>100	-
gastrointestinal panel	**Caco-2**	24h	27.15±0.86[a]	>100	42.58±0.55[b]	190.63±7.68[c]	5.43±0.41[d]
		48h	24.20±0.67[a]	38.38±0.45[b]	38.04±0.52[b]	158.39±8.65[c]	-
	**HT29**	24h	35.74±1.11[a]	>100	69.25±1.79[b]	>100	2.06±0.14[c]
		48h	31.35±1.89[a]	66.51±0.58[b]	60.04±0.92[b]	191.06±6.53[c]	-
	**HepG2**	24h	16.59±0.29[a]	98.00±7.44[b]	126.80±9.60[c]	>100	2.05±0.11[d]
		48h	13.62±0.27[a]	63.05±1.78[b]	107.10±5.44[c]	>100	-
lung panel	**A549**	24h	18.69±1.11[a]	13.76±0.70[b]	150.13±6.19[c]	>100	2.01±0.11[d]
		48h	14.83±0.68[a]	8.58±0.30[a]	73.15±3.34[b]	207.61±22.51[c]	

Abbreviations and symbols: **1**: 3-*O*-β-D-glucopyranosyl(1→3)-β-D-glucopyranosyl] hederagenin, **2**: 3-*O*-β-D-glucopyranosyl(1→3)-β-D-glucopyranosyl] hederagenin 28-*O*-β-D-glucopyranosyl ester, **3**: 28-*O*-β-D-glucopyranosyl hederagenin ester, **4**: hederagenin, DOX: doxorubicin, HTB-140: malignant melanoma, A375: malignant melanoma, WM793: primary melanoma, HaCaT: normal keratinocytes, DU-145: metastatic prostate carcinoma, PC3: metastatic prostate carcinoma, PNT2: prostate epithelial cells, FTC133: follicular thyroid carcinoma, 8505C: undifferentiated thyroid carcinoma, Caco-2: colon adenocarcinoma, HT29: colon adenocarcinoma, HepG2: hepatocellular carcinoma, A549: lung carcinoma

Means in the same row that do not share the letter are significantly different.
